# MicroRNA-206: A Potential Circulating Biomarker Candidate for Amyotrophic Lateral Sclerosis

**DOI:** 10.1371/journal.pone.0089065

**Published:** 2014-02-20

**Authors:** Janne M. Toivonen, Raquel Manzano, Sara Oliván, Pilar Zaragoza, Alberto García-Redondo, Rosario Osta

**Affiliations:** 1 Laboratorio de Genética Bioquímica (LAGENBIO-I3A), Departamento de Anatomía, Embriología y Genética Animal, Universidad de Zaragoza, Zaragoza, Spain; 2 Unidad de ELA, Instituto de Investigación Hospital 12 de Octubre, SERMAS, and Centro de Investigación Biomédica en Red de Enfermedades Raras (CIBERER U-723), Madrid, Spain; National Institute of Health, United States of America

## Abstract

Amyotrophic lateral sclerosis (ALS) is a lethal motor neuron disease that progressively debilitates neuronal cells that control voluntary muscle activity. Biomarkers are urgently needed to facilitate ALS diagnosis and prognosis, and as indicators of therapeutic response in clinical trials. microRNAs (miRNAs), small posttranscriptional modifiers of gene expression, are frequently altered in disease conditions. Besides their important regulatory role in variety of biological processes, miRNAs can also be released into the circulation by pathologically affected tissues and display remarkable stability in body fluids. In a mouse model of ALS that expresses mutated human superoxide dismutase 1 (SOD1-G93A) skeletal muscle is one of the tissues affected early by mutant SOD1 toxicity. To find biomarkers for ALS, we studied miRNA alterations from skeletal muscle and plasma of SOD1-G93A mice, and subsequently tested the levels of the affected miRNAs in the serum from human ALS patients. Fast-twitch and slow-twitch muscles from symptomatic SOD1-G93A mice (age 90 days) and their control littermates were first studied using miRNA microarrays and then evaluated with quantitative PCR from five age groups from neonatal to the terminal disease stage (10–120 days). Among those miRNA changed in various age/gender/muscle groups (miR-206, -1, -133a, -133b, -145, -21, -24), miR-206 was the only one consistently altered during the course of the disease pathology. In both sexes, mature miR-206 was increased in fast-twitch muscles preferably affected in the SOD1-G93A model, with highest expression towards the most severely affected animals. Importantly, miR-206 was also increased in the circulation of symptomatic animals and in a group of 12 definite ALS patients tested. We conclude that miR-206 is elevated in the circulation of symptomatic SOD1-G93A mice and possibly in human ALS patients. Although larger scale studies on ALS patients are warranted, miR-206 is a promising candidate biomarker for this motor neuron disease.

## Introduction

Amyotrophic lateral sclerosis (ALS) is a motor neuron disease that affects 4–6 individuals per 100 000 worldwide, a lifetime risk for developing the condition being approximately one in 400 [1], [2]. ALS leads to degeneration of upper and lower motor neurons. The clinical disease is characterized by progressive skeletal muscle weakness/atrophy and in majority of patients culminates in fatal respiratory paralysis within 3–5 years of the onset of symptoms. There is no cure for ALS and the only approved disease-modifying drug, Riluzole, slows the progression to death only modestly [3]. The identification of ALS is complicated and time consuming as no test can currently establish the disease. The diagnostic process relies on series of clinical examinations combined with electrodiagnostics and other tests to rule out alternative disorders with a similar presentation. Indeed, a significant overlap with some clinically alike conditions at the early stage of ALS considerably delays the progress to the definite diagnosis [4], [5]. While the etiology of ALS at large remains unknown, both environmental and genetic factors are known to be involved. Most cases are sporadic (SALS) and approximately 5% have family history (FALS) [6]. However, the two forms of the disease are clinically indistinguishable. Causes of FALS have been mapped to more than 20 genes [7] and mutations in superoxide dismutase 1 (SOD1), account for approximately one fifth of FALS and a small percentage of apparently sporadic cases.

A transgenic mouse strain overexpressing one of the pathogenic human SOD1 alleles, SOD1-G93A, is a frequently used transgenic model of ALS [8], [9]. Most ALS-linked SOD1 mutations are thought to exert their molecular effects through toxic gain of function(s) by the mutant enzyme [10]. Although the exact nature of the novel molecular properties gained remains elusive, mutant SOD1 (mSOD1) models mimic the main features of ALS and have offered valuable insights to the basic pathology of the disease. One of these is that ALS is not simply a neuronal disease. Neuron-restricted expression of the mSOD1 is not always sufficient to cause the disease [11], [12], although this may depend on neuronal mSOD1 load [13]. Contribution by non-neural cells such as neuroglia, however, is evident [14] and altered function of inhibitory interneurons that monitor and control motor neuron activity may be involved [15]. Furthermore, skeletal muscle-autonomous mSOD1 toxicity strongly contributes to the disease progression in these models; muscle-restricted expression of the mSOD1 is sufficient to trigger ALS symptoms and muscle atrophy [16] as well as motor neuron degeneration [17]. Muscle atrophy in rodent models and ALS patients is also associated with similar perturbations in molecular networks controlling muscle organization and function including autophagy, mitochondrial homeostasis, hypermetabolism and myosatellite cell function [16], [18]–[24]. Therefore, structural and metabolic alterations in cells that connect with neurons, including skeletal muscle, may synergistically exacerbate the disease outcome.

At early stages of ALS pathology, motor neuron cell bodies in the central nervous system remain mostly intact. Motor neuron degeneration initiates by distal withdrawal of nerve terminals from the neuromuscular junction (NMJ) and proceeds retrogradely along the motor axon. This “dying back” pathology is observed in both SOD1-G93A mice and post-mortem samples from human ALS patients [25], [26]. Motor units, functional units of the motor system, consist of a single motor neuron and a group or muscle fibers with similar properties. The denervation in ALS preferentially affects large, fast-conducting motor neurons and, as a result, fast-twitch motor units are more vulnerable to the pathology than slow-twitch units [26]–[29]. The symptomatic stage characterized by tremors and hind-leg spasticity commences in SOD1-G93A mice approximately at postnatal day 90 [8]. However, muscle girth measurements, medical imaging and histological findings all indicate loss of hind-limb muscle volume and decreased fast muscle fiber diameter before the age of 60 days [30], [31]. This suggests early subclinical pathology of skeletal muscle in mSOD1 models.

microRNAs (miRNAs) are evolutionarily conserved non-coding RNA molecules involved in post-transcriptional regulation of gene expression. This regulation is achieved by pairing of miRNA with complementary sequences found in its target mRNAs [32]. The usual consequence of the miRNA:mRNA binding is a downregulation of the corresponding mRNA and/or protein levels due to mRNA destabilization or translational inhibition. Recent evidence gained through simultaneous mRNA and proteomic/ribosomal profiling suggests that the former is dominant in mammalian cells [33]–[35]. miRNAs are frequently altered in conditions such as cancer, cardiovascular and metabolic diseases, and in disorders of central nervous system [36]–[39]. miRNAs are known to be secreted by various cell types and, unlike most mRNAs, are remarkably stable in circulating body fluids due to proteic or vesicular protection from ribonucleases [40]–[42]. Because of these properties miRNAs have recently gained attention for their potential as minimally invasive and cost-effective disease biomarkers.

Here, we first studied by microarrays how miRNA expression is affected in vulnerable (fast) and resistant (slow) muscle types of symptomatic SOD1-G93A mice. The rationale for using skeletal muscle was based on its contribution to the ALS pathology in mice [16], [17] and on the fact that it represents the largest tissue in the human body, possessing a great potential to release miRNAs to the circulation. The selected candidate miRNAs from the microarray data were then investigated individually with quantitative PCR (qPCR) from neonatal, presymptomatic, symptomatic and terminal stage mice in the two muscle types and in the circulation of same model. The relative abundance of the implicated miRNAs was finally measured in serum samples from ALS patients and control individuals. The data indicates that muscle-enriched miR-206 may serve as a non-invasive circulating biomarker for ALS, and warrants larger scale studies on SALS and FALS patients.

## Results

### Muscle miRNA Profile in Symptomatic SOD1-G93A Mice

To gain first insight into the miRNA expression in the skeletal muscle tissue of SOD1-G93A animals, RNA derived from fast extensor digitorum longus (EDL) and slow soleus (SOL) muscles of mutant (denoted +) and control (denoted -) mice at postnatal day P90 (P+days of age) was analysed with miRNA microarrays. It was first assessed how miRNA profile is altered in each muscle type of the mutant compared to the wild type littermates (EDL+ vs. EDL−, and SOL+ vs. SOL−). Secondly, we analysed which miRNA species are significantly more expressed in one of the muscle types in the normal context (EDL− vs. SOL−) and if this enrichment is affected in the mutants (EDL+ vs. SOL+). For main subtypes of probesets found in the arrays were mature miRNA (miRNA), passenger RNA (miRNA*) and stem-loop (pre-miRNA). Some probesets corresponding to non-miRNA species (fragments of tRNA, rRNA, mtDNA etc.) are listed on the Tables S1, S3 and S4 but were not considered as real miRNA changes.

**Figure 1:**
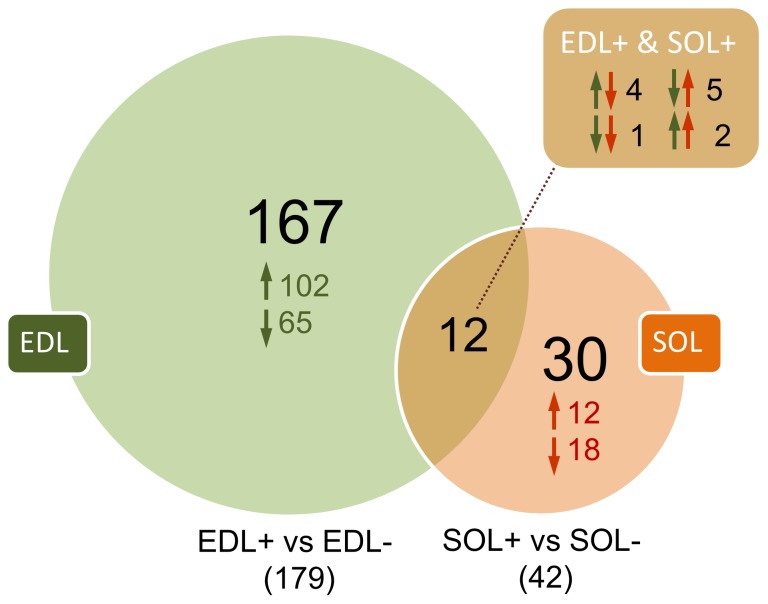
miRNA alterations in the skeletal muscle of symptomatic ALS mice. Venn diagram showing the significant (p<0.05) changes observed in EDL (green, total 179 changes) and SOL (orange, total 42 changes) muscles of SOD1-G93A mutants (+) compared with wild type (−) littermates. Arrows pointing up and down indicate upregulation and downregulation, respectively. An overlap with the two muscle types was observed in 12 probe sets, only three of which were changed in the same direction (brown square). Total 1412 Mus musculus miRNA probe sets were analysed using Affymetrix GeneChip miRNA 2.0 Arrays. Significantly affected non-miRNA species (n = 8), such as fragments of tRNA, rRNA and the mitochondrial genome, are not included in the data but are listed in Tables S1 and S2.

Without multiple corrections (see Materials and methods), comparison of SOD1-G93A with controls revealed 179 significant (p<0.05) changes in EDL+ (108 up, 71 down), and 42 changes in SOL+ (19 up, 23 down). Only 12 probesets were common among those affected in EDL+ and SOL+ muscles, leaving 167 mutant EDL-specific changes and 30 changes specific to mutant SOL muscle (Figure 1, see also clustering of the affected probesets in Figure S1). The fold changes associated with SOD1-G93A expression in the EDL varied from 11.9 fold upregulation to −4.2 fold downregulation and in SOL from 2.7 fold upregulation to −2.6 fold downregulation. The most upregulated mature miRNA in the mutant EDL were on average more highly expressed (7.8±0.6 fold) than most upregulated miRNA* (3.6±0.6 fold, p<0.001) or pre-miRNA (1.9±0.3 fold, p<0.001) species, whereas most downregulated mature miRNA did not significantly differ from that of miRNA* or pre-miRNA (Figure S2). In EDL, 56 probesets were upregulated and 10 probesets downregulated more than two fold, most (79%) of those being mature miRNAs (Figure S3). In SOL, only one probeset was up and one down more than two fold (both mature miRNAs). The complete lists of significant changes in SOD1-G93A EDL and SOL compared to the respective wild type muscles are shown in Tables S1 and S2, respectively.

Comparison of the fast and slow muscle type within wild type animals (EDL− vs. SOL−) revealed total 118 probesets with preferential expression in one of the muscle types (Table S3). Of these, approximately one half were preferentially expressed in EDL and the other half in SOL. Of those 31 probesets that were enriched more than 2 fold in one of the muscles, 12 was primarily expressed in EDL and 19 in SOL. Similar comparison with the two muscle types in SOD1-G93A mutants (EDL+ vs. SOL+, Table S4) revealed total 77 significant changes, 43 of which were preferentially EDL expressed and 34 preferentially SOL expressed. Eight probesets were enriched more than 2 fold in one of the muscle types (4 in EDL and 2 in SOL) in the mutants.

One quarter (25%) of the total probesets up-regulated in the mutant EDL muscle (EDL+ vs. EDL−) were coincident with those preferentially expressed in wild type SOL (EDL− vs. SOL−), most (84%) of these being SOL enriched for more than 2 fold. Respectively, one third (32%) of the total downregulated miRNA in the mutant EDL muscle (EDL+ vs. EDL−), were preferably expressed in EDL of the controls (EDL− vs. SOL−). As expected, multiple corrections by false discovery rate (FDR), which indicates the fraction of false positives within a list of genes exceeding a given statistical cutoff (see materials and methods fo details) markedly reduced the number of significant changes in the miRNA list. In fact only two miRNA from the EDL+ vs. EDL− comparison remained significant (q-value<0.05) after FDR correction, and none from SOL+ vs. SOL− comparison. The most promising miRNA candidates were then shortlisted for individual assays considering following criteria: 1) published evidence supporting that the target is real miRNA, 2) preference for mature miRNAs, 3) preference for miRNA affected in EDL, 4) preference for high/moderate array signal intensities to low ones, 5) preference for higher fold change and most significant p-values and, 6) those miRNA implicated in muscle biology. Total 24 miRNA were tested first for sufficient expression level in EDL, SOL and plasma from wild type and mutant animals. A limit where miRNA is considered expressed in sufficient level for reliable analysis was set to 32 PCR cycles at least in one of the muscles and plasma (Figure S4). Sufficiently high levels of expression was found only in 10 miRNAs: miR-133a, miR-206, miR-1, miR-145, miR-24, miR-19b, miR-17, miR-106b, miR-20a and miR-21. Additionally, miR-133b that was not altered in the arrays was selected for its known function in muscle biology. Some of the miRNA were found to be expressed in very low amounts in muscle and not detectable in plasma. Such included some of the most upregulated miRNA in the array data, such as miR-379, miR-671-5p and miR-708 (Figure S4).

### Disease-stage Affects miRNA Alterations in SOD1-G93A Skeletal Muscle and Blood Plasma

The selected 11 miRNAs were examined in various postnatal stages that in SOD1-G93A mice correspond to neonatal (P10), early presymptomatic (P40), late presymptomatic (P60), symptomatic (P90) and terminal phase (P120). Individual miRNAs were measured from EDL and plasma of male mice from all age groups, and from EDL and plasma of females from P60, P90 and P120. As soleus is known to be affected later in SOD1-G93A model, SOL was tested only at two oldest age groups for both sexes. Briefly, the only miRNA whose expression was increased consistently at any stage/tissue/sex was miR-206. The other significantly altered miRNAs (miR-1, -133a, -133b, -145, -21 and miR-24) were always found to be diminished, although modestly (≤50%) and varying between the age and muscle/plasma/sex groups. All observed miRNA alterations in the two muscles and plasma of SOD1-G93A animals are listed in Table 1.

**Table 1:** Relative* miRNA alterations verified by qPCR in SOD1-G93A animals.

Tissue	miRNA	P60	p (P60)	P90	p (P90)	P120	p (P120)
EDL	miR-206	1.7 (M)	0.1	**7.7 (M)/7.7 (F)**	0.0001/0.001	**11.7 (M)/4.2 (F)**	0.0001/0.03
	miR-1	–	–	0.5 (F)	0.1	**0.4 (F)**	0.05
	miR-133a	–	–	**0.6 (F)**	0.004	–	–
	miR-133b	–	–	–	–	**0.7 (F)**	0.04
	miR-106b	–	–	2.3 (M)	0.11	–	–
Plasma	miR-206	3.8 (M)	0.1	**4.5 (M)/**3.3 (F)	0.0004/0.09	**0.6 (M)/4.2 (F)**	0.04/0.02
	miR-1	–	–	–	–	**0.2 (M)**	0.001
	miR-133a	–	–	–	–	**0.3 (M)/**2.6 (F)	0.005/0.1
	miR-133b	–	–	–	–	**0.3 (M)**	0.01
	miR-145	–	–	1.7 (M)	0.12	**0.5 (M)**	0.04
	miR-21	–	–	–	–	**0.5 (M)**	0.05
	miR-24	–	–	–	–	**0.5 (M)**	0.03
	miR-106b	2.3 (M)	0.1	–	–	–	–
SOL	miR-206	N/A	N/A	–	–	–	–
	miR-1	N/A	N/A	0.7 (M)	0.1	**0.5 (M)/**0.6 (F)	0.002/0.06
	miR-133a	N/A	N/A	**0.8 (M)**	0.01	–	–
	miR-133b	N/A	N/A	0.7 (M)	0.1	–	–
	miR-145	N/A	N/A	**0.7 (M)/**0.6 (F)	0.02/0.1	0.7 (M)/0.6 (F)	0.08/0.01

Significant results (p<0.05) shown in bold, those close to significance (0.05<p>0.15) in normal font.

*Relative numbers >1 refer to upregulation, and those <1 to downregulation. Abbreviations: M, males; F, females; p(PX), p-value at indicated postnatal day X.

**Figure 2:**
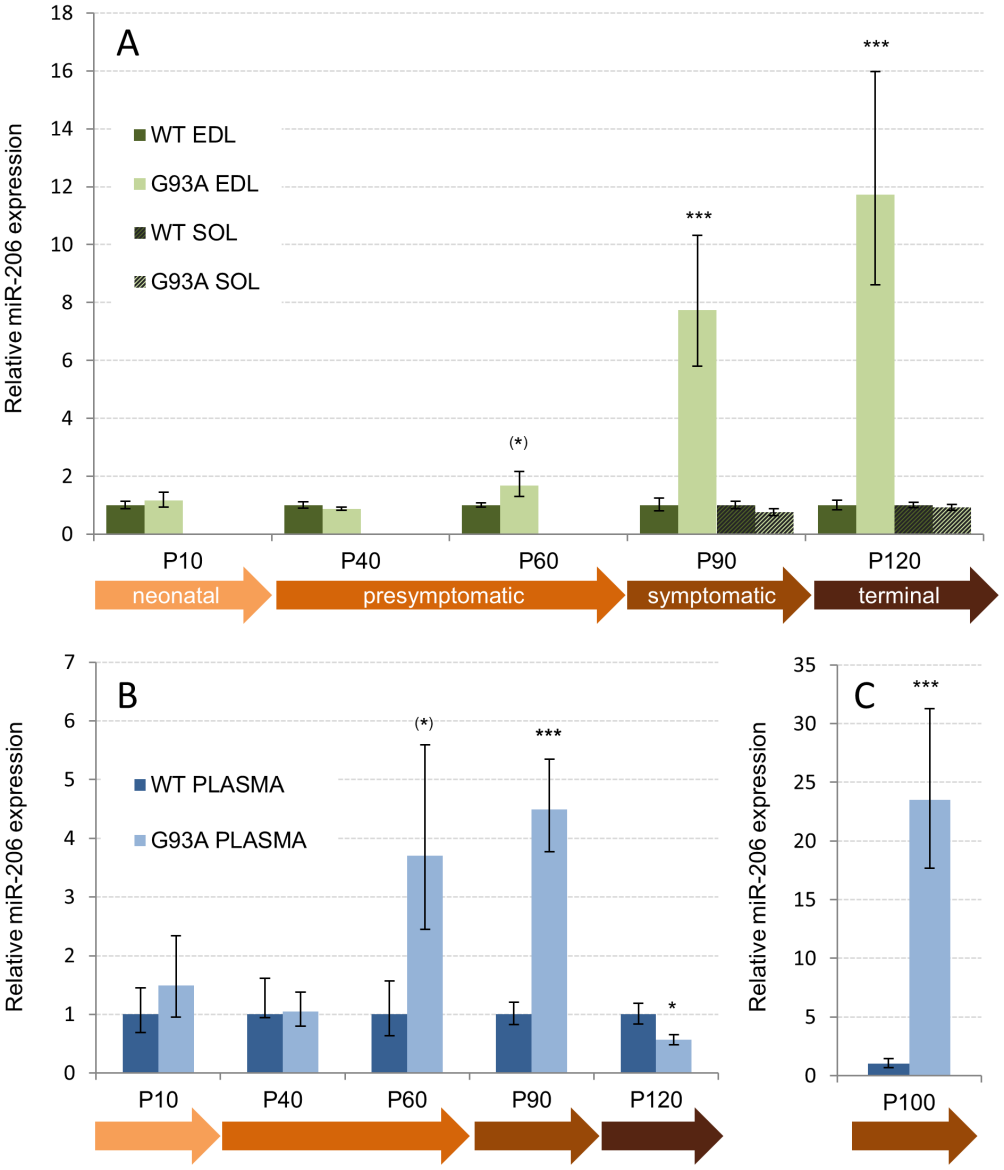
miR-206 is elevated in fast-twitch muscle and circulation of SOD1-G93A males. A) Relative miR-206 expression in male SOD1-G93A muscles at various stages of the pathology (P10– P120). Wild type (set as 1) is shown as dark green, SOD1-G93A as light green. EDL is shown as solid bars, SOL as hatched bars. B) Relative miR-206 expression in male SOD1-G93A plasma. Wild type (set as 1) is shown as dark blue, SOD1-G93A as light blue. C) Relative miR-206 expression in SOD1-G93A plasma from symptomatic animals at postnatal day 100. The expression values are relative to the age-matched controls obtained by 2^−ΔΔCt^ method and the error bars are those obtained after 2^−ΔΔCt^ conversion of standard deviation (see materials and methods). ^(*)^p = 0.1 (close to significance), ^*^p<0.05 (significant), ^***^p<0.001(highly significant).

In SOD1-G93A males, no changes in any of the studied miRNA were found at P10, P40 or P60, although suggestive but non-significant upregulation of miR-206 was observed at P60 in EDL (Figure 2A, 1.7 fold, p = 0.10) and plasma (Figure 2B, 3.8 fold, p = 0.10). At symptomatic P90, miR-206 was 7.7 fold upregulated in EDL (p = 0.0001), 4.5 fold upregulated in plasma (p = 0.0004). At terminal P120, miR-206 was 11.7-fold upregulated in male EDL muscle (p = 0.0001) but, perhaps surprisingly, showed a −40% decrease in circulating plasma (p = 0.04). miR-206 was unaffected in slow SOL muscle at P90 or P120 (Figure 2A, bars with diagonal stripes). Albeit the relative levels of miR-206 in male EDL increased progressively towards the end stage, the circulating miR-206 dropped dramatically from the onset of symptoms to the terminal stage (Figures 2A and 2B). Because it remained possible that the peak elevation in circulating miR-206 has been missed between the age groups P90 and P120, miR-206 was further studied from male mice at the age of 100–105 days (mean 102 days in controls and 101.9 days in mutants). Indeed, plasma miR-206 was upregulated 23.5 fold (p<0.00001, Figure 2C) at this age.

**Figure 3:**
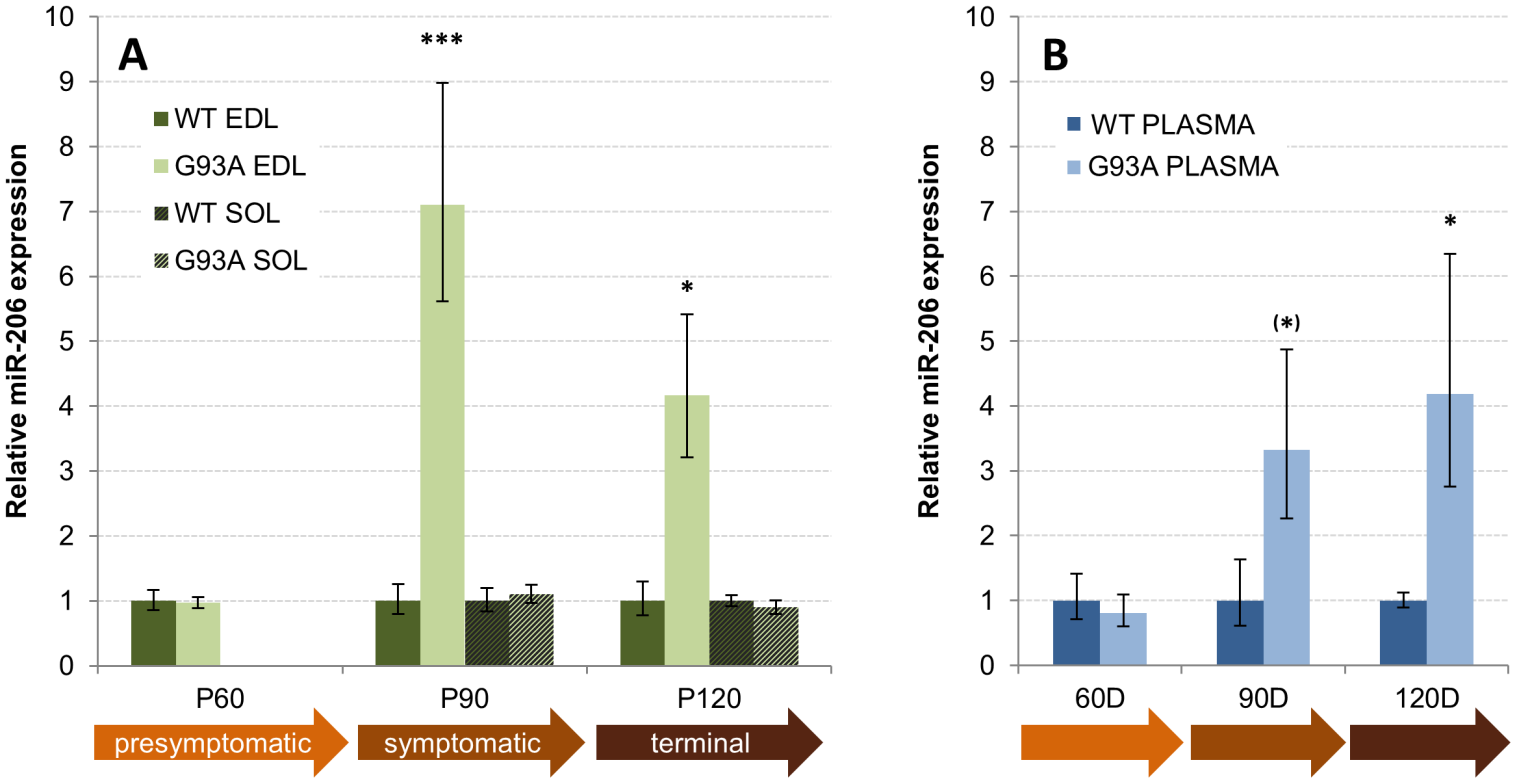
miR-206 is elevated in fast-twitch muscle and circulation of SOD1-G93A females. A) Relative miR-206 expression in female SOD1-G93A muscles at various stages of the pathology (P60– P120). B) Relative miR-206 expression in female SOD1-G93A plasma. See text and Figure 2 for details.

In SOD1-G93A females (Figure 3), like in males, miRNA levels were unchanged at P60 EDL (Figure 3A) and plasma (Figure 3B). At P90, miR-206 was 7.1 fold increased in EDL (p = 0.0001, Figure 3A) and its upregulation was suggestive but non-significant in P90 plasma (3.3 fold, p = 0.09, Figure 3B). At P120, miR-206 was 4.2 fold up in female EDL (p = 0.03) to the same extent in circulating plasma (p = 0.02). Like in males, miR-206 was unaffected in SOL (Figure 3A, bars with diagonal stripes).

### Circulating miRNAs in ALS Patients

**Figure 4:**
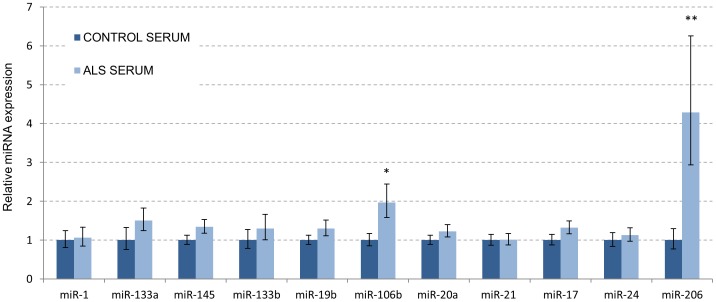
miR-206 and miR-106b are elevated in the circulation of ALS patients. Relative expression of 11 miRNAs studied in definitive ALS patients (n = 12) vs. healthy controls (n = 12). See text for details. ^*^p<0.05 (significant), ^**^p<0.01(very significant).

As one of the main objectives of the study was to find out candidate miRNA biomarkers for ALS, a small group of patient serum samples was analysed for above mentioned 11 miRNAs. Serum from six male and six female patients with definite ALS (El Escorial Criteria, [43]) and from six healthy male and six healthy female controls were studied. Average age of the patients was 57±12.2 years, and that of controls 54±14.5 years. Genders separated the average age of males was 57±17.9 years for patients and 50±15.7 years for controls, and for females 58±10.0 and 58±9.1 years, respectively. Both genders combined, the qPCR analysis (Figure 4) indicated 4.3 fold upregulation of miR-206 (p = 0.005) and 2 fold upregulation of miR-106b (p = 0.02) in patient versus control serum samples. Because we reasoned that some changes may be modified by sex, we also compared the data within gender groups. Comparison of male patients with male controls did not result in significant changes, although miR-206 was close to significance (3.4 fold upregulation, p = 0.1, Figure S5A). Comparison of female patients with female controls resulted in significant 5.4 fold upregulation of miR-206 (p = 0.02), 2-fold upregulation of miR-133b (p = 0.03) and 1.4 fold upregulation of miR-145 (p = 0.04) (Figure S5B).

## Discussion

The study examined miRNA alterations in the skeletal muscle and circulation of SOD1-G93A model of ALS, and used the obtained results to conduct the first investigation of miRNA alterations in the circulation of ALS patients. The most outstanding result is that levels of miR-206, which is known to be involved in the maintenance of neuromuscular connectivity in ALS mice [44], are elevated not only in the affected muscle of both male and female SOD1-G93A mice but also in the blood plasma of these animals and in serum samples from human ALS patients.

The number of miRNA alterations in the hybridization data (Figures 1 and S1) is in agreement with fast-twitch muscles being more affected in SOD1-G93A model than slow-twitch muscles [26]–[29]. As postnatal skeletal muscles can adapt by switching fiber type depending on motoneuron activity [45], preferential motor unit loss and reinnervation of the fast fibers by surviving motor units leads to a fast-to-slower fiber type transition in SOD1-G93A mice [46]. EDL of mice consist mainly of type IIB and IIDB fibers, whereas SOL is composed mainly of type I, IIA and IIAD fibers [47]. Interestingly, a large number of miRNAs that in control animals were enriched in the slow SOL muscle were here upregulated in fast EDL muscle of SOD1-G93A (upward arrows in Table S3). In contrast, many of those normally enriched in fast muscle showed diminished expression in SOD1-G93A EDL (downward arrows, Table S3). This could suggest that fiber type transition described in SOD1-G93A fast-twitch muscle is reflected by the corresponding fast-to-slow transition in the miRNA profile, and that the regulated miRNA may have a role in the determination of fiber type or associated metabolic switch. However, it is equally possible that the mSOD1 associated miRNA alterations are just reflecting the fiber type transition without playing active part in the process. The latter is supported by the observation that the two known miRNAs involved in fast-to-slow fiber programs, miR-208b and miR-499 [48], were not affected in SOD1-G93A model (although miR-208b was found to be slow-twitch enriched in both mutant and control animals, Tables S3 and S4).

Of those miRNAs ultimately selected for qPCR verification, several were under detection limit in one or both muscles, and some most upregulated miRNAs were absent in circulation. One of the indications of the presented mouse work is that the microarray data should be interpreted with caution, especially when the signal intensities are low. Although microarrays are useful in screening large number of potential miRNA candidates and, therefore, creating lists of potentially altered miRNA for further work, it may be misleading to publish these data without confirmation using more accurate methods.

When all ages are combined, the only consistently altered miRNA in the course of ALS pathology in SOD1-G93A mice was miR-206. The increase in the levels of miR-206 was first significant in the symptomatic stage, consistent with its upregulation in denervated tibialis anterior (TA) muscle and in symptomatic TA muscle of a similar ALS model [44]. miR-206 expression is controlled by myogenic regulatory transcription factors including myogenin and MyoD that are also upregulated by denervation [49]–[51]. Upregulation of miR-206 in male EDL and plasma was close to significance at presymptomatic stage, which suggests that the threshold of fast muscle denervation at which miR-206 expression is induced is crossed between postnatal days 60 and 90, where also the myogenic factors involved in miR-206 transcription are induced [23]. miR-206 and miR-133b are clustered, and their co-expression as bicistronic transcript in myogenic conversion in vitro and in denervation in vivo has been documented [44], [50]. Here, however, miR-206 upregulation in SOD1-G93A mice was not associated with miR-133b upregulation at any stage. Similar results have been obtained in Duchenne muscular dystrophy model (mdx) suggesting that in some conditions the two miRNAs are under independent transcriptional control [52].

Elevated circulating miR-206 levels were detected in both sexes of SOD1-G93A mice, although the level and age at which this occurred differed according to the symptomatic onset (males are affected earlier in this model). Furthermore, although miR-206 levels in male EDL muscle progressively increased towards the terminal stage (Figure 2A), its circulating levels (Figure 2B) were decreased at the terminal stage where muscles are severely atrophic. Therefore, although severe muscle atrophy could dampen miR-206 expression directly, this is not supported at least in males where the most severely damaged EDL shows the highest miR-206 expression. However, the release or secretion of miRNAs from the muscle tissue to the circulation, including that of miR-206, may be a regulated process that requires uncompromised muscle function which is lost at the terminal disease stage (between P100 and P120). This conclusion may be supported by the general finding that seven out of 11 tested miRNAs were significantly downregulated in the circulation of terminal stage males (Table 1). Discrepancies between miRNA expression between the muscle tissue and serum have been also described in mdx mice, where many myomirs are highly abundant in plasma without alterations in the muscle [53]–[55]. However, it cannot be excluded that other factors influencing miRNA stability in the circulation may be affected.

Many miRNAs are expressed in a tissue-specific manner. Myomirs are miRNAs whose expression is enriched in striated and/or cardiac muscle, and are involved in myogenesis, developmental muscle growth and cardiac function [56]. Increase in circulating myomirs miR-1, miR-133a, miR-133b and miR-206 have been demonstrated in various models of striated muscle pathologies [41], [53], [57] whereas they are frequently downregulated in cancer [58]. Although miR-206 is the only myomir whose expression was described to be striated muscle-specific, it has subsequently been shown to be expressed also in brown fat [59], in liver [60], in subset of helper T-cells [61], and in breast tissue where its expression may be under hormonal control [62]. miR-206 is also elevated in the nervous system in various pathological conditions including Alzheimer’s disease and cerebral ischemia [63], [64], in schizophrenia [65], as well as in cytotoxic insult upon exposure to environmental toxin [66]. This may indicate pathological induction of miR-206 in the neuronal or associated cells where it is not normally expressed. Alternatively, muscle-derived circulating miRNAs may be actively transported to other cell types via extracellular vesicles, where they may possibly act as posttranscriptional regulators [40].

miR-206 is important for differentiation of myoblasts as it downregulates of several inhibitors myogenesis [67]. On the other hand, it seems to be dispensable for maintaining post-natal skeletal muscle mass [68]. Deletion of miR-206 in vivo does not lead to muscle atrophy or alterations in NMJ maturation. Instead, miR-206 upregulation is required for formation of new NMJs after nerve injury and contributes to the capability to maintain neuromuscular connections in SOD1-G93A animals *in vivo* by potentiating the function of muscle-derived factors on NMJ regeneration [44]. Besides the protective role of miR-206 in SOD1-G93A model, its other potential functions in ALS remain unknown. Among the thousands of miR-206 target transcripts predicted by various bioinformatics tools [69] more than 50 are experimentally validated (Table 2). These include a large number of factors involved in transcriptional regulation (transcription factors and those involved in associated chromatin modifications), cell cycle and signaling, secreted factors and receptors, as well as proteins that function in synaptic or cell junctions. Because miRNA target validation methodology relies largely on in vitro assays these factors may or may not have a direct role in tissues contributing to ALS pathogenesis. Although many forms of posttranscriptional regulation are not obvious from RNA-level studies, it is noticeable that transcripts for nine of the listed targets (BDNF, CCND2, IGFBP5, NFAT5, NOTCH3, OTX2, SH3BGRL3, TIMP3, and TPPP) are downregulated in muscle biopsies from ALS patients ([70], EBI-EMBL Expression Atlas ID E-GEOD-3307). For example, IGFBP5 is a positive regulator of IGF1 (also target of miR-206, Table 2) signalling and both are severely reduced in ALS muscle [71]. Very little is known about the other miRNA implicated in ALS patient samples, miR-106b. It is known to be expressed from a conserved cluster including three miRNAs (mir-106b, mir-93 and miR-25) that are involved in cancer [72] and adult neural stem cell proliferation and differentiation [73]. Additionally, miR-106b has been implicated in brown adipocyte differentiation [74] and in suppression of autophagy in cultured myoblasts [75]. Whether miR-206 and miR-106b have a functional role in human ALS pathogenesis needs further experimental focus, preferably using methodology that can reveal miRNA target genes and pathways directly in the affected tissues.

**Table 2:** Experimentally verified targets for miR-206.

				Evidence				
Process	Gene symbol	Gene product function	Experimental cells	LUC	WB	qPCR	NB	Refs
Transcription/chromatin	ACTL6A	Subunit of SWI/SNF complex	HEK293, NIH10T1/2	•	•			[79]
	CLOCK	bHLH transcription factor	indirect evidence					[56], [80]
	ESR1	estrogen hormone receptor	MCF-7, MDA-MB-231	•	•	•		[62]
	HDAC4	histone deacetylase	COS1, miR-206-KO	•	•			[44], [68], [81]
	HIF1A*	hypoxic transcriptional activator	PASMC	•	•	•		[82]
	HMGB3	high mobility group box protein	HEK293	•		•		[83]
	KLF4**	transcription factor	HMEC, RK3E, MCF10A	•		•		[84], [85]
	MEOX2	homeobox transcription factor	NIH3T3, DF1	•	•			[67]
	MSC	transcriptional repressor	C2C12				•	[86]
	NCOA1 & NCOA3	nuclear receptor coactivators	MCF-7		•	•		[87]
	NFAT5	transcription factor	NIH3T3, DF1	•	•			[67]
	OTX2	homeobox transcription factor	U343, SK-N-SH	•	•	•		[88]
	PAX3	paired box transcription factor	HEK293, C2C12, SMSC	•	•			[89]–[92]
	PAX7	paired box transcription factor	SMSC, C2C12,	•	•	•		[52], [93]–[95]
	RARB	retinoic acid receptor	C2C12, NIH3T3, DF1	•	•			[67]
	SMARCB1	SWI/SNF transcriptional co-activator	C2C12, NIH3T3, DF1	•	•			[67]
	SMARCD2	SWI/SNF transcriptional co-factor	C2C12, NIH3T3, DF1	•	•			[67]
	SNAI2	transcriptional repressor	SMSC, COS7		•	•		[96]
Cell signaling	HHIP	hedgehog signalling inhibitor	C2C12				•	[86]
	MAP4K3	MAP protein kinase	C2C12, NIH3T3, DF1	•	•			[67]
	NRP1	Cell migration signalling	AML12			•		[97]
	PRICKLE1	Wnt/beta-catenin signaling	zebrafish embryo		•			[98]
	PTPLAD1	Rac1 signaling	C2C12	•			•	[86]
Cell cycle	CCND1 & CCND2	G1/S-specific cyclins	RMS, GC, C2C12, HeLa	•	•	•	•	[92], [99], [100]
	CDC42	small GTPase of the Rho-subfamily	MDA-MB-231		•			[101]
	ID1, ID2 & ID3	dominant negative HLH proteins	C2C12				•	[86]
	POLA1	DNA replication	C2C12	•			•	[86]
Secretedfactor	BDNF	growth factor	C2C12, HEK293T, N2a	•	•	•	•	[86], [102]–[104]
	FSTL1	glycoprotein of the follistatin family	MEF	•			•	[105]
	IGF1	growth factor	HEK293	•				[106]
	IGFBP5	IGF binding protein (stimulatory)	miR-206-KO SMSC	•	•	•		[95]
	TAC1	peptide hormone/neurotransmitter	MSC, MSC-NC	•		•		[107]
	VEGFA	growth factor	LSCC		•			[108]
Membranereceptor	FZD7	Wnt signaling receptor	C2C12, NIH3T3, DF1	•	•			[67]
	MET	hepatocyte growth factor receptor	RMS, SMSC	•	•			[109], [110]
	NGFR	nerve growth factor receptor	C2C12	•		•		[103]
	NOTCH3	Notch signalling receptor	HeLa, MEFs, SMSC	•	•	•		[95], [111], [112]
Synapse/junction	GJA1	gap junction protein	C2C12, mouse	•	•		•	[67], [86], [113]
	IGSF5	tight junction adhesion molecule	C2C12				•	[86]
	UTRN	NMJ and cytoskeleton component	MEF	•	•	•		[105]
Others	CLCN3	endosome/synaptic vesicle antiporter	C2C12, NIH3T3, DF1	•	•			[67]
	GPD2	mitochondrial dehydrogenase	AML12			•		[97]
	MMD	monocyte to macrophagedifferentiation	C2C12	•	•		•	[86]
	MUP1	glucose and lipid metabolism	AML12			•		[97]
	SH3BGRL3	TNF inhibitory protein	DF1	•				[67]
	TIMP3	metalloproteinase inhibitor	CF	•	•	•		[114]
	TPPP	microtubule network organizer	CG4, HeLa	•				[115]
	FN1	cell adhesion, migration	H441	•	•	•		[116]

*Hif1a upregulation in protein level, downregulation in mRNA level and LUC assay, **miR-206 can stimulate (normal cells) or repress (cancer cells) KLF4 translation. Abbreviations: LUC, luciferase assay; WB, western blot; qPCR, quantitative PCR; NB, northern blot; HEK293, human embryonic kidney cells; NIH10T1/2, fibroblasts; MCF-7, breast cancer cell line; MDA-MB-231, human adenocarcinoma cells; miR-206-KO,miR-206 knockout mouse; PASMC, pulmonary artery smooth muscle cells; HMEC, human mammary epithelial cells; RK3E, rat kidney cells; MCF10A, mammary epithelial cells; NIH3T3, mouse embryonic fibroblast cells; DF1, chicken embryonic fibroblasts; U343, human glioma cells; SK-N-SH, human neuroblastoma cells; C2C12, immortalized mouse skeletal myoblasts; SMSC, mouse skeletal muscle satellite cells; RuGli, rat glioma cells; COS7, African green monkey kidney cells; AML12, mouse hepatocytes; RMS, rhabdomyosarcoma cells; GC, human gastric cancer cells; HEK293T, human embryonic kidney cells with SV40 Large T-antigen; N2a, mouse neuroblastoma cells; SH-SY5Y, human neuroblastoma cells; MEF, mouse embryonic fibroblasts; MSC, human mesenchymal stem cells; MSC-NC, human mesenchymal stem cell-derived neural cells; HeLa, human epithelial cells from cervical carcinoma; CF, cardiac fibroblasts; CG, rat oligodendrocyte progenitor cells; H441, human lung adenocarcinoma epithelial cells.

Because of mostly sporadic nature, rapid progression and clinical heterogeneity of ALS, an early detection by biomarkers affected in ALS but not other conditions that mimic the disease would certainly be welcome in the community. Neurophysiological and imaging techniques may be used to help disease monitoring and get insight on disease prospects [76]. Additionally, higher concentrations of phosphorylated neurofilament heavy subunit (pNF-H) and cystatin C seem to be associated with more rapid functional decline and survival in ALS patients, and could therefore serve as potential prognostic biomarkers for the disease [76]. However, many of these have been mainly assessed in cerebrospinal fluid, and those obtainable by blood or other accessible body fluids (urine, saliva etc.) would be desirable. The two miRNAs found to be elevated here, miR-206 and miR-106b, may provide ideal biomarkers as they can be sampled from blood. Without doubt, more samples from ALS patients need to be analysed to draw definitive conclusions about miRNA changes, and other possible miRNA changes are warrant screening to increase the power of the test. As the miR-206 profile in ALS mice is profoundly altered depending on the severity of pathological stage, it will be necessary to characterize the miRNA changes in a larger number of patients involving samples from possible, probable and definite ALS patient groups, as well as separating the groups with bulbar and limb onset as well as demographic factors etc. Importantly, a comparative study of circulating miRNAs between ALS patients and disorders that mimic ALS need to be conducted. This is crucial especially for the diagnostic purposes, as with rare diseases the number of false positives may greatly exceed the number of affected cases if the specificity of a biomarker is not close to 100%. Recent data on mdx model of Duchenne muscular dystrophy suggest that serum levels of several Myomirs can be used as biomarkers of muscle turnover [41]. In this model, the elevated circulating levels of miR-1, miR-133a and miR-206 show dose-responsive restoration to wild type levels in response to exon-skipping therapy that restores dystrophin levels. Therefore, it may be plausible that miR-206 (and miR-106b) can be used in the future to screen potential candidate drugs or treatments for ALS.

In conclusion, the increased circulating miR-206 and miR-106b may serve as biomarkers for ALS in humans. Further studies are warranted to investigate the diagnostic or prognostic value of these miRNAs within different subtypes of ALS as well as within suspected population including ALS-like conditions. Importantly, it will be of interest to evaluate the applicability of these miRNAs as indicators of therapeutic response in pre-clinical testing and in clinical trials.

## Materials and Methods

### Ethics Statement

All experimental animal procedures were approved (Ref. PI31/10) by the Ethics Committee of Universidad de Zaragoza and followed the international and the institutional guidelines for the use of laboratory animals. Existing human serum samples were from ALS Unit of Instituto de Investigación Biomédica Hospital 12 de Octubre, Madrid, Spain. They were stored in frozen state until the study and were analysed anonymously. The present study has been approved by the ethics committee “Comité ético de investigación clínica – CEIC” del Instituto de Investigación Biomédica del Hospital 12 de Octubre “i+12″. We have obtained written informed consent of every patient included in this study.

### Animals and Housing

The transgenic mice B6SJL-Tg(SOD1-G93A)1Gur/J [8] expressing a high copy number of the G93A mutant form of human SOD1 were obtained from the Jackson Laboratory (Bar Harbor, ME, USA) and were housed under a 12 h light: 12 h dark cycle in 21–23°C with relative humidity of 55%. Food and water were available *ad libitum*. Transgenic animals were maintained by breeding hemizygous SOD1-G93A males with B6SJL wild type females. The genotype was determined from a tail sample as described (http://www.jax.org/). Hemizygous SOD1-G93A mice and their non-transgenic littermates were used for all experiments. At the terminal stage, SOD1-G93A mice that were unable to right themselves within 20 seconds when placed on their side were sacrificed for ethical reasons.

### Tissue Extraction

The blood was extracted from CO_2_-euthanized animals, after which a cervical dislocation was performed before the muscle dissection. Briefly, 0.4 ml blood was extracted from the heart using sterile K3-EDTA –treated syringe with 25 gauge needle. Blood was slowly transferred to a microcentrifuge tube containing 10 µl K3-EDTA and centrifuged at 1300×g for 10 minutes at 4°C and 120 µl plasma was collected without interference with the lower cell fraction. Plasma samples were frozen within 30 minutes of the sacrifice and stored at −80°C. Extensor digitorum longus (EDL) and soleus (SOL) muscles were dissected immediately after the blood extraction and submerged each in 0.5 ml RNAlater® (Life Technologies, Cat# AM7021). The samples were stored at +4°C for 24 hours and then transferred to −80°C.

### RNA Extraction

For microarrays, muscle tissues stored frozen in RNAlater were pulverized in a mortar under liquid nitrogen. The total RNA was extracted using mirVana miRNA Isolation Kit (Life Technologies, Cat# AM1560) according to kit instructions. Each RNA sample was eluted in 100 µl of RNase free water. The quality and quantity of each extraction was confirmed by Bioanalyzer ® (Agilent Technologies) and Nanodrop ND-1000 spectrophotometer (Thermo Scientific). The mean RNA integrity (RIN) number of the samples was 9.1 (range 8.6–9.5). For qPCR, the individual muscles stored frozen in RNAlater were homogenized in Cellcrusher cryogenic tissue pulverizer (Cellcrusher, Cork, Ireland) submerged in liquid nitrogen. The frozen muscle powder was transferred to a microcentrifuge tube containing 0.5 ml QIAzol lysis reagent (Qiagen, Cat# 79306), then further processed by passing 10 times through a 23 gauge needle and centrifuged 12000×g for 1 minute. Without disturbing the insolubilized pellet, 0.45 ml Trizol/muscle lysate was transferred into a new tube and RNA was purified using Direct-zol RNA miniprep (Zymo Research, Cat# R2052) following manufacturer’s instructions. The samples were eluted in 50 µl RNase free water and stored at −80°C for further analysis. Plasma samples (each 120 µl) were thawn on ice and re-centrifuged at 1300×g for 5 minutes at 4°C. Total plasma RNA (including miRNA) from exactly 100 µl of plasma was extracted with Norgen Total RNA Purification kit (Norgen Biotek Corporation, Cat# 17200) with minor modifications. Briefly, after inactivation of RNases with lysis solution, carrier RNA (0.56 µg MS2 RNA, Roche, cat# 10165948001) and spike-in miRNA (25 fmol synthetic cel-miR-39, Qiagen, Cat# MSY0000010) was added for increased consistency of the RNA isolation and for normalization, respectively. The plasma lysate was passed through provided spin columns and washed 3 times with absolute ethanol. RNA was eluted in 50 µl elution solution by successive 2-minute centrifugations at 200×g and 14 000×g and then immediately stored at −80°C. The RNA from human serum samples were prepared as above, except starting from 200 µl serum.

### Microarrays

The microarray hybridization of microRNAs was performed by Progenika Biopharma, Spain, using GeneChip ® miRNA 2.0 chips (Affymetrix®). These contain total 1412 mouse probesets (722 mature miRNA and 690 pre-miRNA species) that cover those miRNAs included in miRBase v15 (ftp://mirbase.org/pub/mirbase/15/). Left EDL and left SOL muscle from three SOD1-G93A males and three age matched male littermates (total 12 samples) were analysed at the symptomatic age of 90 days. From each sample, 1 µg of total RNA was used as a starting material. Sample processing and hybridization, development, scanning of the chips and analysis of the results was carried out following the protocols and equipment recommended by Affymetrix Inc. The software used for processing chips and the results was the Affymetrix GeneChip Command Console Software v3.0 (SCFA 3.0, Affymetrix ®) and miRNA QCTool Software (Affymetrix ®). The software used for statistical analysis of the results was Partek® Genomics Suite™ and dChip (www.dchip.org) [77]. Briefly, the probe intensity values were background corrected, quantile-normalized and the probeset-level expression signals were summarized with the robust multi-array average (RMA) method. The significant differences (p-value >0.05, tables S1–S4) in each muscle/genotype comparison were first determined by t-test. Because individual p-values for each probeset are not the best measure of significance in the context of testing large number of miRNAs, we then calculated the confidence of microarray results using the false discovery rate (FDR). This approach controls the number of false discoveries in “significantly altered” probesets and determines adjusted p-values (FDR q-values, tables S1–S4) for each test. Only two probesets were found significant (q-value <0.05) after FDR. Therefore, the miRNAs for individual assays were selected weighing significance and the expression fold-change, as well as other criteria explained in “Results”. The microarray data in its completion will be available for public access in NCBI Gene Expression Omnibus (GEO, www.ncbi.nlm.nih.gov/geo).cDNA synthesis.

From each muscle RNA sample, 30 ng was used as a template for RT with TaqMan® MicroRNA Reverse Transcription Kit (Life Technologies, Cat #4366596). For plasma samples, where RNA quantification was not feasible, 5.35 µl spiked-in plasma RNA was directly used as a template. The standard TaqMan® MicroRNA Assays protocol calls for an individual RT reaction for each target miRNA. To facilitate the miR-cDNA synthesis, multiplexed RT step was used that included pools of individual RT primers. RT primer pools were prepared as instructed by the manufacturer (Life Technologies). Multiplex ‘23X-pool’ was prepared by mixing 23 individual TaqMan miRNA assays of interest (those listed in Figure S4, excluding miR-206), as well as those of snoRNA-202 or cel-miR-39 for normalization of muscle and plasma samples, respectively (see below). For miR-206, RT pool ‘206-pool’ was prepared separately with snoRNA-202 or cel-miR-39 as the interference of miR-206 with highly homologous miR-1 did not allow these RT primers to be included in the pool. Significant results derived from the pooled RT reactions were later confirmed using individual RT reactions.

### Quantitative PCR

Individual TaqMan® miRNA assays (Life Technologies) were used to quantify the muscle and plasma-expressed miRNAs. The muscle samples were normalized with snoRNA202. As no endogenous circulating miRNA with proven suitability for qPCR normalization currently exist, equal amount of plasma was used as starting material. To allow normalization for potential variability in the RNA extraction step the plasma samples were spiked in with 25fmol synthetic *C. elegans* miR-39 (cel-miR-39) which does not have orthologs in mammalian species. Each condition (genotype, sex, age) contained minimum 6 biological replicates (animals). qPCR from each sample was run in three technical replicates. For sample normalization, mean cycle threshold (Ct) values of snoRNA202 (for muscle) or cel-miR-39 for (spiked-in plasma/serum) were subtracted from the mean Ct values or the miRNA under study (ΔCt). Within same sex and age, the mean of control animal ΔCt values were then subtracted from the mean of mutant animal ΔCt values (ΔΔCt). The exponential process was converted to the linear relative comparison by 2^−ΔΔCt^ conversion. The control group in each comparison was used as a calibrator (ΔΔCt = 0, 2^−ΔΔCt^ = 1). The positive and negative error values (error bars) were also determined using 2^−ΔΔCt^ method [78]. Statistical analysis was performed by means of Student’s t-test, where values were considered statistically significant at P<0.05.

## Supporting Information

Figure S1 **Heatmap of hierarchical cluster analysis of microarray samples.** Summarized intensity values from the differentially expressed miRNAs in EDL and SOL muscles of wild type (−) and SOD1-G93A (+) animals are shown. Data includes 187 and 42 probesets from tables S1 and S2 respectively. The log2 intensity values are shown in the bar scale. Hierarchical dendrograms are shown above the heatmaps and the numbers indicated (12SEXXX) denote the sample IDs for EDL− (12SE849-51), EDL+ (12SE852-54), SOL- (12SE855-57) and EDL+ (12SE858-60).
(TIF)

Figure S2 **Highly upregulated probesets are mostly mature miRNA.** The ten most overexpressed mature miRNAs are more upregulated than those corresponding to miRNA* or pre-miRNA species. Average relative expression values (EDL+ vs EDL−) of ten most upregulated (red) and ten most down regulated (green) probesets are shown for mature miRNAs (miRNA), passenger strand (miRNA*) and pre-miRNAs. The error bars are standard error of mean (SEM). ^***^p<0,001, ^**^<0,01.
(TIF)

Figure S3 **The probesets affected more than 2-fold in SOD1-G93A EDL muscle.** Average relative expression values (EDL+ vs EDL−) of probesets upregulated or downregulated at least 2-fold are shown. Blue bars represent mature miRNAs, red bars miRNA* species, and green bars pre-miRNAs. The identities of the specific Affymetrix probesets are indicated on the X-axis. See text for details.
(TIF)

Figure S4 **Determining the detection limits for qPCR.** A) Cutoff values (Ct) for 24 miRNAs were determined from EDL (blue bars), SOL (red) and plasma (green) samples. Cutoff for significant expression was set to 32 (dashed red line), at least in plasma and one of the muscles (EDL or SOL). B) Numerical data for graph shown in A. The assays selected for the final qPCR verification are indicated in column “selected”.
(TIF)

Figure S5 **Data for** **Figure 4** **genders separated.** A) Relative serum levels of 11 miRNAs studied in definitive ALS male patients (n = 6) vs. healthy control males (n = 6). B) Relative serum levels of 11 miRNAs studied in definitive ALS female patients (n = 6) vs. healthy control females (n = 6). See text for details. ^(*)^p<0.01(close to significance), ^*^p<0.05 (significant).
(TIF)

Table S1 **Microarray changes in SOD1-G93A EDL muscle compared with wild type EDL.** All significant changes without multiple corrections are listed. Positive fold change (FC) indicates higher expression in the SOD1-G93A mutants, and negative FC lower higher expression in the wild type animals. Probeset ID refers to the Affymetrix probeset identifier.
(PDF)

Table S2 **Microarray changes in SOD1-G93A SOL muscle compared with wild type SOL.** All significant changes without multiple corrections are listed. Positive fold change (FC) indicates higher expression in the SOD1-G93A mutants, and negative FC lower higher expression in the wild type animals. Probeset ID refers to the Affymetrix probeset identifier.
(PDF)

Table S3 **Preferential expression of microarray probesets in wild type EDL vs wild type SOL.** All significant changes without multiple corrections are listed. Positive fold change (FC) indicates preferential (higher) expression in the wild type EDL, and negative FC lower preferential expression in the wild type SOL. Probeset ID refers to the Affymetrix probeset identifier.
(PDF)

Table S4 **Preferential expression of microarray probesets in SOD1-G93A EDL vs SOD1-G93A SOL.** All significant changes without multiple corrections are listed. Positive fold change (FC) indicates preferential (higher) expression in the SOD1-G93A EDL, and negative FC lower preferential expression in the SOD1-G93A SOL. Probeset ID refers to the Affymetrix probeset identifier.
(PDF)
