# Supplementary figures and images for: MicroRNA-206: A Potential Circulating Biomarker Candidate for Amyotrophic Lateral Sclerosis
Source: PLoS One. 2014 Feb 20;9(2):e89065. doi: 10.1371/journal.pone.0089065 (PMC3930686; doi:10.1371/journal.pone.0089065)

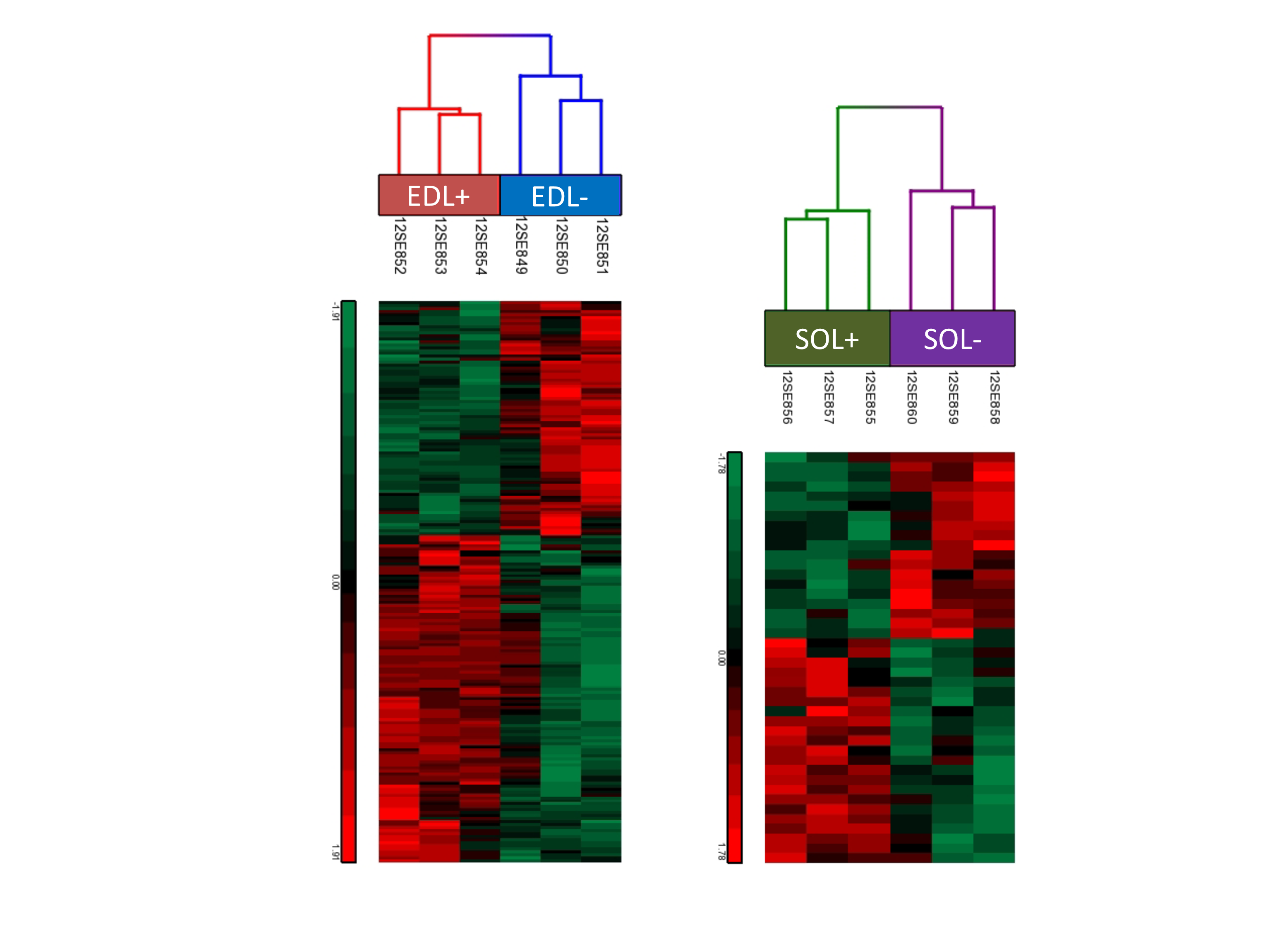

Supplement: Figure S1 — Heatmap of hierarchical cluster analysis of microarray samples. Summarized intensity values from the differentially expressed miRNAs in EDL and SOL muscles of wild type (−) and SOD1-G93A (+) animals are shown. Data includes 187 and 42 probesets from tables S1 and S2 respectively. The log2 intensity values are shown in the bar scale. Hierarchical dendrograms are shown above the heatmaps and the numbers indicated (12SEXXX) denote the sample IDs for EDL− (12SE849-51), EDL+ (12SE852-54), SOL- (12SE855-57) and EDL+ (12SE858-60). (TIF) [file pone.0089065.s001.tif]

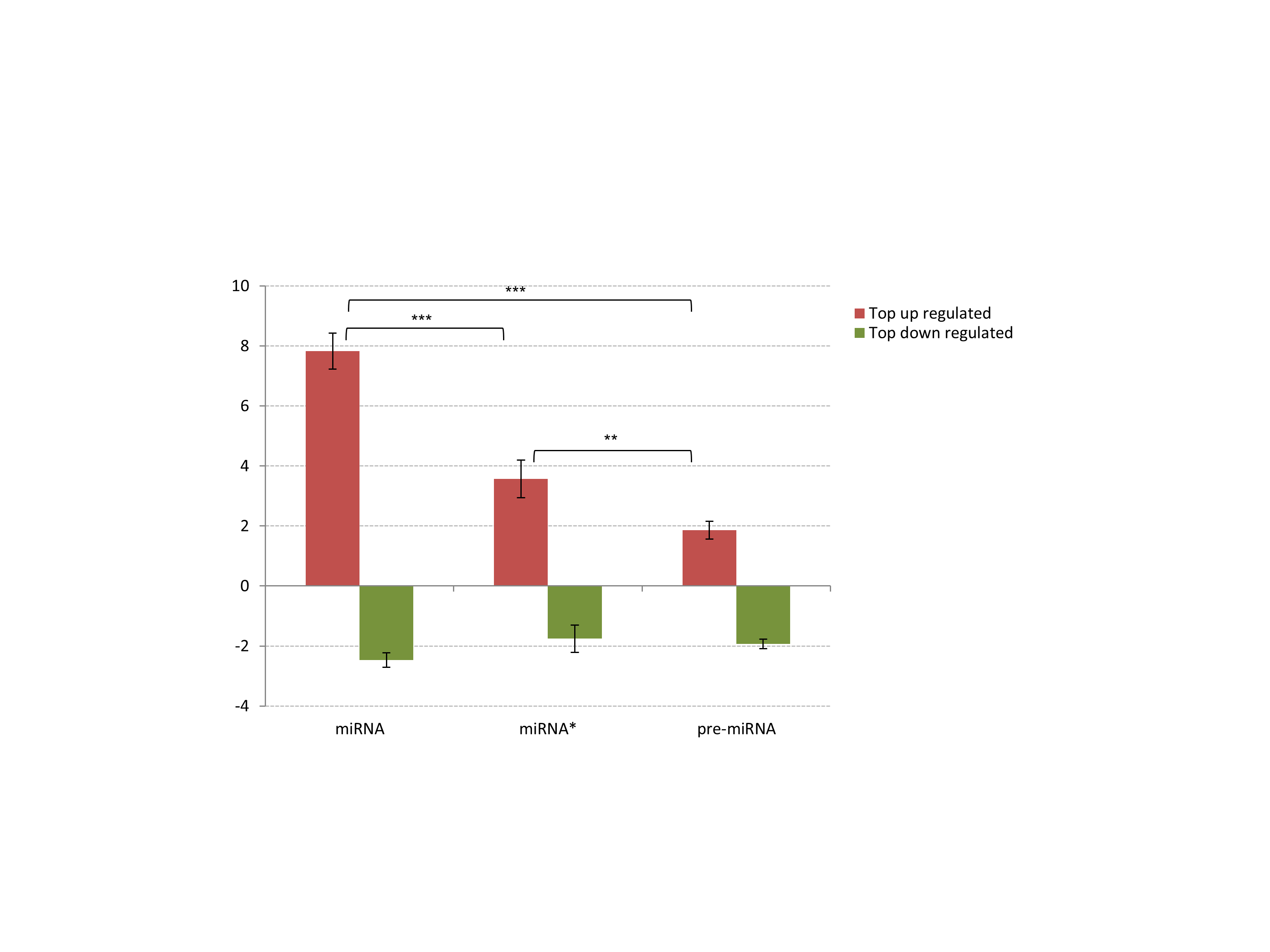

Supplement: Figure S2 — Highly upregulated probesets are mostly mature miRNA. The ten most overexpressed mature miRNAs are more upregulated than those corresponding to miRNA* or pre-miRNA species. Average relative expression values (EDL+ vs EDL−) of ten most upregulated (red) and ten most down regulated (green) probesets are shown for mature miRNAs (miRNA), passenger strand (miRNA*) and pre-miRNAs. The error bars are standard error of mean (SEM). ***p<0,001, **<0,01. (TIF) [file pone.0089065.s002.tif]

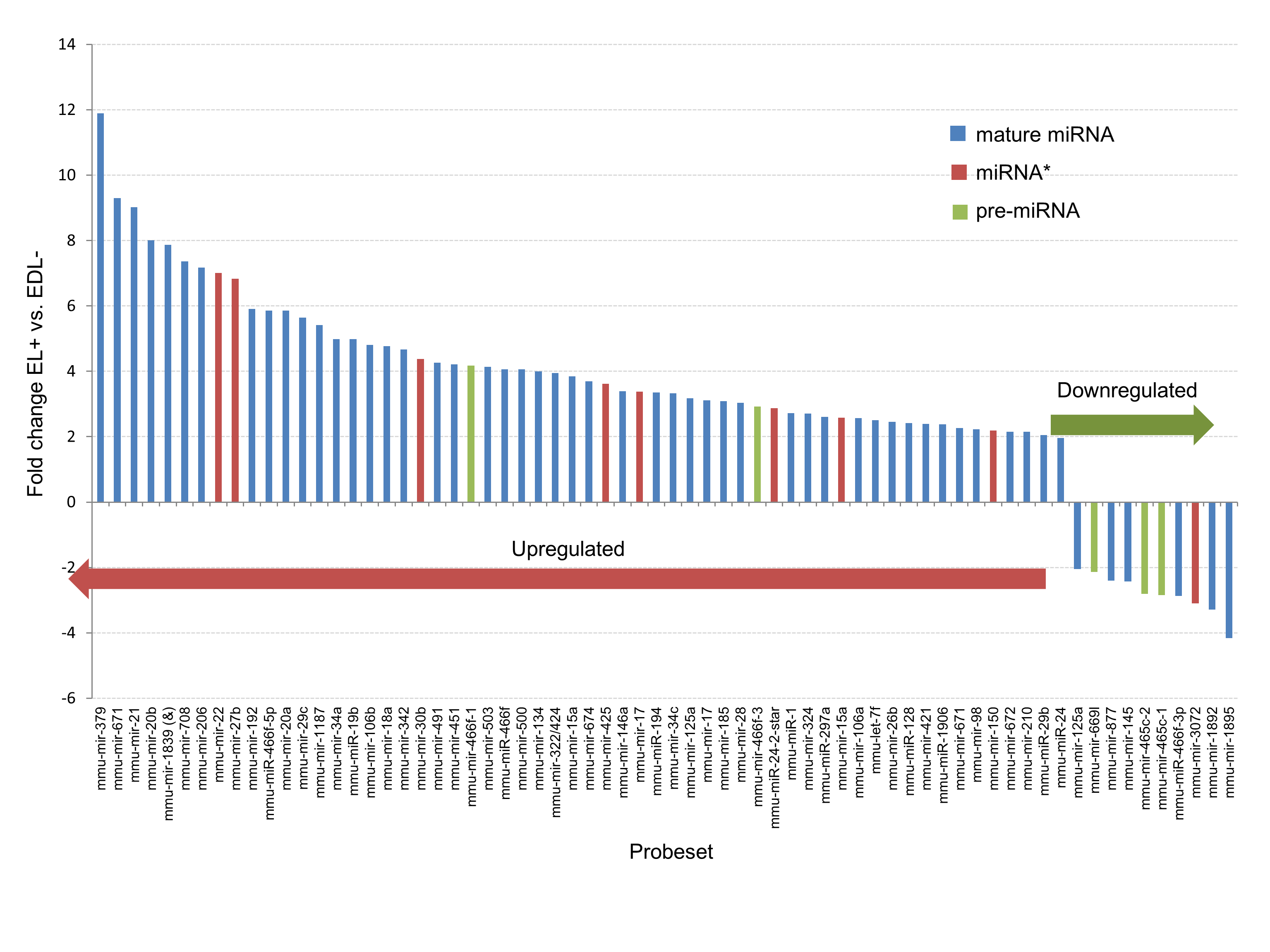

Supplement: Figure S3 — The probesets affected more than 2-fold in SOD1-G93A EDL muscle. Average relative expression values (EDL+ vs EDL−) of probesets upregulated or downregulated at least 2-fold are shown. Blue bars represent mature miRNAs, red bars miRNA* species, and green bars pre-miRNAs. The identities of the specific Affymetrix probesets are indicated on the X-axis. See text for details. (TIF) [file pone.0089065.s003.tif]

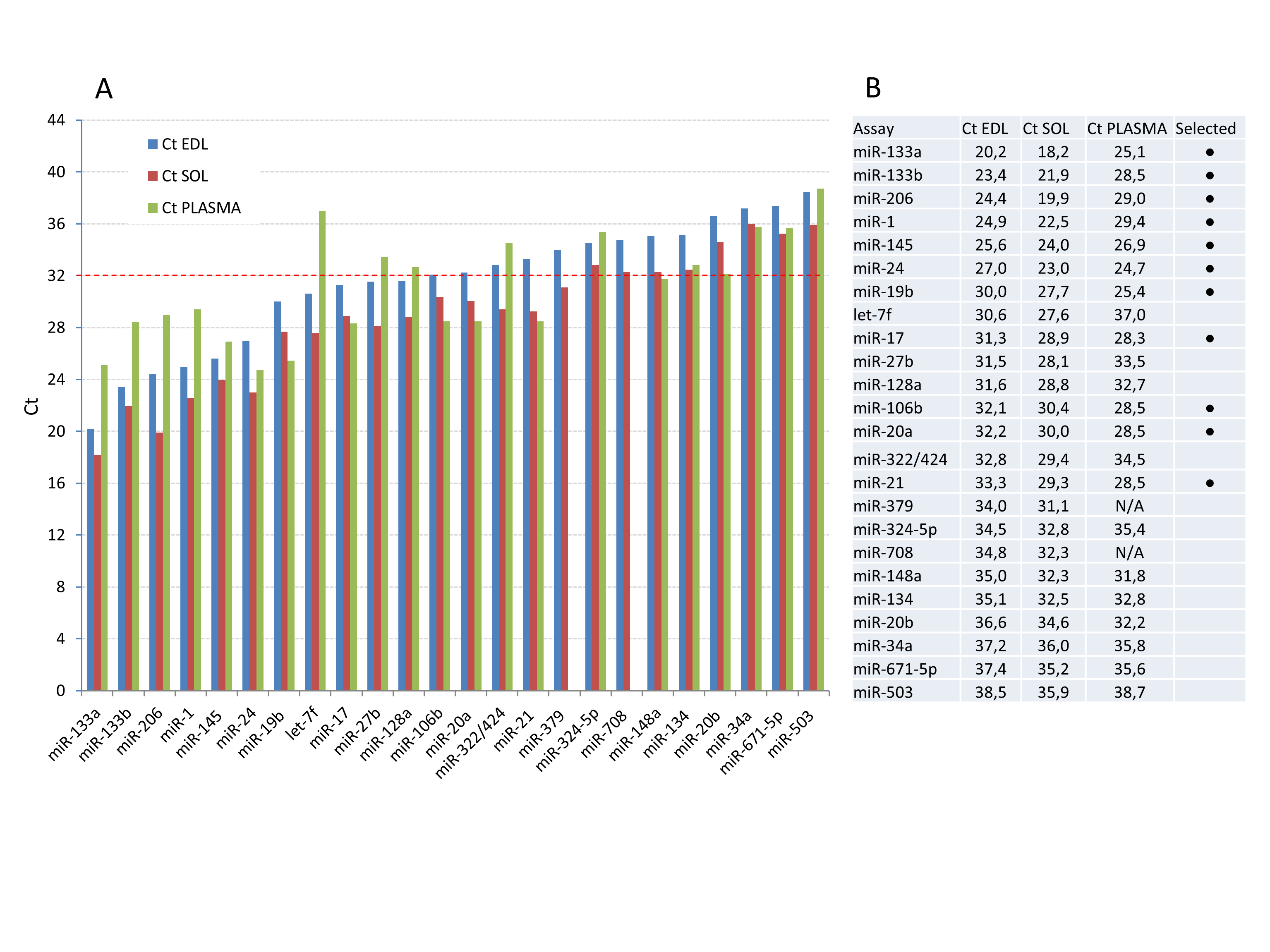

Supplement: Figure S4 — Determining the detection limits for qPCR. A) Cutoff values (Ct) for 24 miRNAs were determined from EDL (blue bars), SOL (red) and plasma (green) samples. Cutoff for significant expression was set to 32 (dashed red line), at least in plasma and one of the muscles (EDL or SOL). B) Numerical data for graph shown in A. The assays selected for the final qPCR verification are indicated in column “selected”. (TIF) [file pone.0089065.s004.tif]

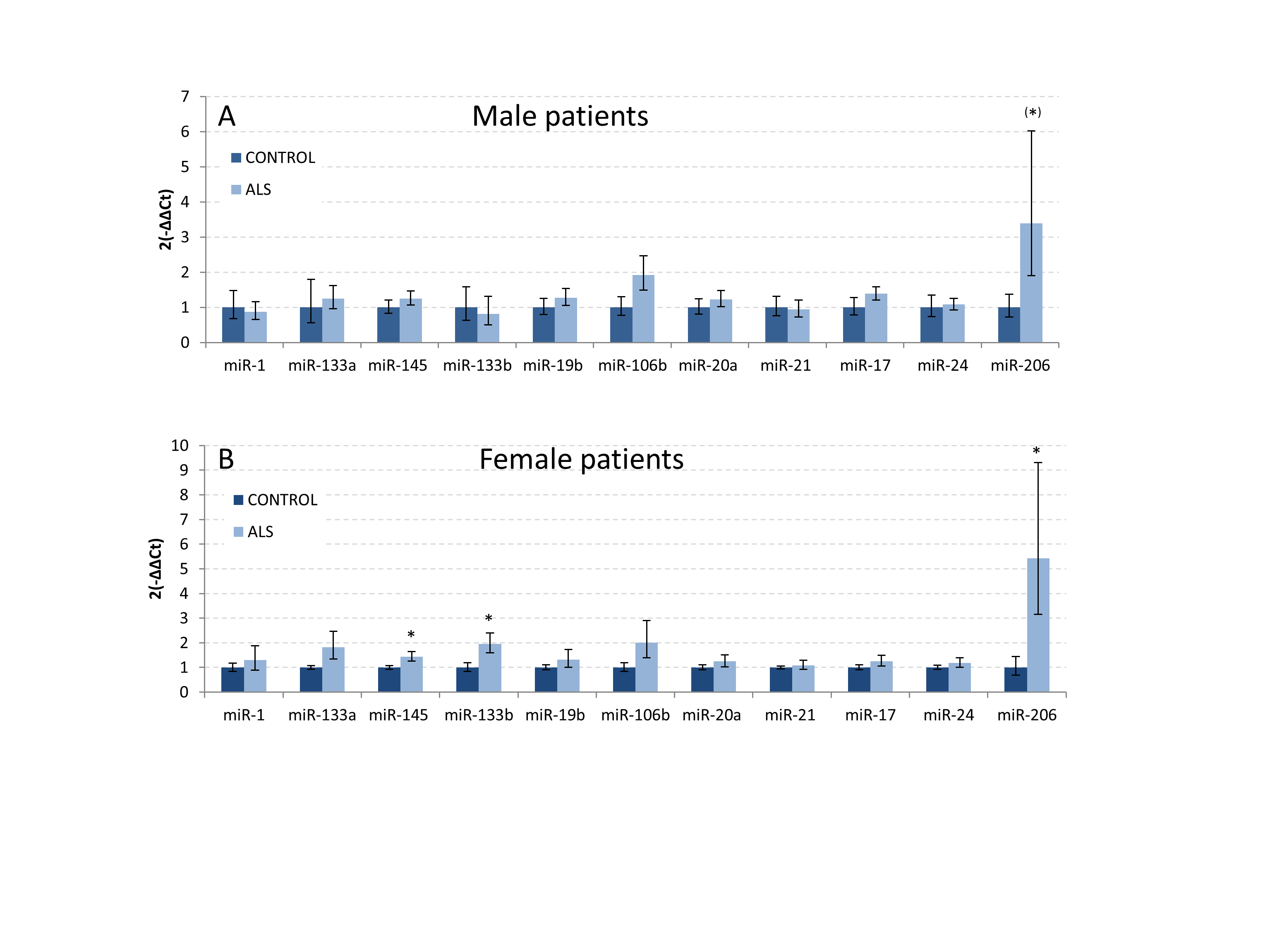

Supplement: Figure S5 — Data for Figure 4 genders separated. A) Relative serum levels of 11 miRNAs studied in definitive ALS male patients (n = 6) vs. healthy control males (n = 6). B) Relative serum levels of 11 miRNAs studied in definitive ALS female patients (n = 6) vs. healthy control females (n = 6). See text for details. (*)p<0.01(close to significance), *p<0.05 (significant). (TIF) [file pone.0089065.s005.tif]
