# Supplementary material for: MicroRNA-206: A Potential Circulating Biomarker Candidate for Amyotrophic Lateral Sclerosis
Source: PLoS One. 2014 Feb 20;9(2):e89065. doi: 10.1371/journal.pone.0089065 (PMC3930686; doi:10.1371/journal.pone.0089065)
Supplement: Table S1 — Microarray changes in SOD1-G93A EDL muscle compared with wild type EDL. All significant changes without multiple corrections are listed. Positive fold change (FC) indicates higher expression in the SOD1-G93A mutants, and negative FC lower higher expression in the wild type animals. Probeset ID refers to the Affymetrix probeset identifier. (PDF) [file pone.0089065.s006.pdf]

Transcript ID	FC (EDL+ vs. EDL-)	p-value	FDR(q-value)	Sequence Type	Probeset ID
mmu-mir-379	11,9	0,0259	0,3116	miRNA	mmu-miR-379_st
mmu-mir-671	9,3	0,0000	0,0239	miRNA	mmu-miR-671-5p_st
mmu-mir-21	9,0	0,0185	0,2961	miRNA	mmu-miR-21_st
mmu-mir-20b	8,0	0,0000	0,0239	miRNA	mmu-miR-20b_st
mmu-mir-1839 (&)	7,9	0,0364	0,3478	miRNA/snoRNA	mmu-miR-1839-5p_st
mmu-mir-708	7,4	0,0007	0,1704	miRNA	mmu-miR-708_st
mmu-mir-206	7,2	0,0001	0,0610	miRNA	mmu-miR-206_st
mmu-mir-22	7,0	0,0090	0,2899	miRNA*	mmu-miR-22-star_st
mmu-mir-27b	6,8	0,0154	0,2961	miRNA*	mmu-miR-27b-star_st
mmu-mir-192	5,9	0,0201	0,2961	miRNA	mmu-miR-192_st
mmu-miR-466f-5p	5,9	0,0049	0,2737	miRNA	mmu-miR-466f-5p_st
mmu-mir-20a	5,9	0,0101	0,2899	miRNA	mmu-miR-20a_st
mmu-mir-29c	5,6	0,0010	0,1704	miRNA	mmu-miR-29c_st
mmu-mir-1187	5,4	0,0183	0,2961	miRNA	mmu-miR-1187_st
mmu-mir-34a	5,0	0,0177	0,2961	miRNA	mmu-miR-34a_st
mmu-miR-19b	5,0	0,0250	0,3116	miRNA	mmu-miR-19b_st
mmu-mir-106b	4,8	0,0098	0,2899	miRNA	mmu-miR-106b_st
mmu-mir-18a	4,8	0,0373	0,3491	miRNA	mmu-miR-18a_st
mmu-mir-342	4,7	0,0217	0,3009	miRNA	mmu-miR-342-5p_st
mmu-mir-30b	4,4	0,0298	0,3168	miRNA*	mmu-miR-30b-star_st
mmu-mir-491	4,3	0,0056	0,2737	miRNA	mmu-miR-491_st
mmu-mir-451	4,2	0,0011	0,1704	miRNA	mmu-miR-451_st
mmu-mir-466f-1	4,2	0,0097	0,2899	stem-loop	hp_mmu-mir-466f-1_x_st
mmu-mir-503	4,1	0,0253	0,3116	miRNA	mmu-miR-503_st
mmu-miR-466f	4,1	0,0136	0,2961	miRNA	mmu-miR-466f_st
mmu-mir-500	4,1	0,0087	0,2899	miRNA	mmu-miR-500_st
mmu-mir-134	4,0	0,0296	0,3168	miRNA	mmu-miR-134_st
mmu-mir-322/424	3,9	0,0083	0,2899	miRNA	mmu-miR-322_st
mmu-mir-15a	3,8	0,0119	0,2961	miRNA	mmu-miR-15a_st
mmu-mir-674	3,7	0,0283	0,3146	miRNA	mmu-miR-674_st
mmu-mir-425	3,6	0,0173	0,2961	miRNA*	mmu-miR-425-star_st
mmu-mir-146a	3,4	0,0490	0,3731	miRNA	mmu-miR-146a_st
mmu-mir-17	3,4	0,0052	0,2737	miRNA*	mmu-miR-17-star_st
mmu-miR-194	3,4	0,0209	0,2977	miRNA	mmu-miR-194_st
mmu-mir-34c	3,3	0,0444	0,3631	miRNA	mmu-miR-34c_st
mmu-mir-125a	3,2	0,0212	0,2990	miRNA	mmu-miR-125a-3p_st
mmu-mir-17	3,1	0,0051	0,2737	miRNA	mmu-miR-17_st
mmu-mir-185	3,1	0,0271	0,3116	miRNA	mmu-miR-185_st
mmu-mir-28	3,0	0,0384	0,3569	miRNA	mmu-miR-28_st
mmu-mir-466f-3	2,9	0,0069	0,2748	stem-loop	hp_mmu-mir-466f-3_x_st
mmu-miR-24-2-star	2,9	0,0167	0,2961	miRNA*	mmu-miR-24-2-star_st
mmu-miR-1	2,7	0,0149	0,2961	miRNA	mmu-miR-1_st
mmu-mir-324	2,7	0,0369	0,3478	miRNA	mmu-miR-324-5p_st
mmu-miR-297a	2,6	0,0016	0,2233	miRNA	mmu-miR-297a_st
mmu-mir-15a	2,6	0,0076	0,2764	miRNA*	mmu-miR-15a-star_st
mmu-mir-106a	2,6	0,0322	0,3300	miRNA	mmu-miR-106a_st
mmu-let-7f	2,5	0,0363	0,3478	miRNA	mmu-let-7f_st
mmu-mir-26b	2,5	0,0169	0,2961	miRNA	mmu-miR-26b_st
mmu-miR-128	2,4	0,0199	0,2961	miRNA	mmu-miR-128_st
mmu-mir-421	2,4	0,0099	0,2899	miRNA	mmu-miR-421_st
mmu-miR-1906	2,4	0,0435	0,3631	miRNA	mmu-miR-1906_st
mmu-mir-671	2,3	0,0230	0,3088	miRNA	mmu-miR-671-3p_st
mmu-mir-98	2,2	0,0412	0,3631	miRNA	mmu-miR-98_st
mmu-mir-150	2,2	0,0099	0,2899	miRNA*	mmu-miR-150-star_st
mmu-mir-672	2,2	0,0257	0,3116	miRNA	mmu-miR-672_st
mmu-mir-210	2,1	0,0359	0,3478	miRNA	mmu-miR-210_st
mmu-miR-29b	2,1	0,0170	0,2961	miRNA	mmu-miR-29b_st
mmu-miR-24	2,0	0,0033	0,2737	miRNA	mmu-miR-24_st
mmu-mir-1941	1,9	0,0112	0,2961	miRNA	mmu-miR-1941-5p_st
mmu-mir-101b	1,9	0,0128	0,2961	miRNA	mmu-miR-101b_st
mmu-mir-598	1,8	0,0202	0,2961	miRNA	mmu-miR-598_st
mmu-mir-669n	1,8	0,0074	0,2764	miRNA	mmu-miR-669n_st
mmu-mir-93	1,7	0,0173	0,2961	miRNA	mmu-miR-93_st

mmu-mir-27b	1,7	0,0163	0,2961	miRNA	mmu-miR-27b_st
mmu-mir-148a	1,7	0,0035	0,2737	miRNA	mmu-miR-148a_st
mmu-mir-574	1,6	0,0450	0,3631	miRNA	mmu-miR-574-5p_st
mmu-mir-615	1,6	0,0101	0,2899	miRNA	mmu-miR-615-5p_st
mmu-mir-466h	1,5	0,0247	0,3116	stem-loop	hp_mmu-mir-466h_x_st
mmu-mir-496	1,5	0,0124	0,2961	miRNA	mmu-miR-496_st
mmu-mir-1954	1,5	0,0070	0,2748	miRNA	mmu-miR-1954_st
mmu-mir-1186b	1,5	0,0260	0,3116	stem-loop	hp_mmu-mir-1186b_x_st
mmu-mir-128-2	1,5	0,0069	0,2748	stem-loop	hp_mmu-mir-128-2_x_st
mmu-mir-485	1,4	0,0455	0,3631	miRNA*	mmu-miR-485-star_st
mmu-mir-122	1,4	0,0314	0,3254	stem-loop	hp_mmu-mir-122_st
mmu-mir-501	1,4	0,0495	0,3731	stem-loop	hp_mmu-mir-501_st
mmu-mir-770	1,4	0,0094	0,2899	miRNA	mmu-miR-770-3p_st
mmu-mir-21	1,4	0,0165	0,2961	miRNA*	mmu-miR-21-star_st
mmu-mir-466j	1,4	0,0285	0,3147	stem-loop	hp_mmu-mir-466j_x_st
mmu-mir-1937a (#)	1,4	0,0188	0,2961	stem-loop/tRNA	hp_mmu-mir-1937a_st
mmu-mir-193	1,4	0,0171	0,2961	stem-loop	hp_mmu-mir-193_st
mmu-mir-1896	1,4	0,0365	0,3478	miRNA	mmu-miR-1896_st
mmu-mir-194-2	1,4	0,0364	0,3478	stem-loop	hp_mmu-mir-194-2_st
mmu-mir-23a	1,4	0,0120	0,2961	stem-loop	hp_mmu-mir-23a_x_st
mmu-mir-107	1,4	0,0357	0,3478	miRNA	mmu-miR-107_st
mmu-mir-23a	1,4	0,0139	0,2961	stem-loop	hp_mmu-mir-23a_st
mmu-mir-204	1,4	0,0280	0,3146	miRNA	mmu-miR-204_st
mmu-mir-291b	1,4	0,0263	0,3116	stem-loop	hp_mmu-mir-291b_st
mmu-mir-686	1,3	0,0254	0,3116	stem-loop	hp_mmu-mir-686_s_st
mmu-mir-183	1,3	0,0169	0,2961	miRNA	mmu-miR-183_st
mmu-mir-883a	1,3	0,0443	0,3631	miRNA	mmu-miR-883a-3p_st
mmu-mir-467e	1,3	0,0075	0,2764	miRNA	mmu-miR-467e_st
mmu-mir-34b	1,3	0,0058	0,2737	miRNA	mmu-miR-34b-5p_st
mmu-mir-291a	1,3	0,0405	0,3631	stem-loop	hp_mmu-mir-291a_x_st
mmu-mir-17	1,3	0,0173	0,2961	stem-loop	hp_mmu-mir-17_st
mmu-mir-323	1,3	0,0314	0,3254	stem-loop	hp_mmu-mir-323_st
mmu-miR-16	1,3	0,0497	0,3731	miRNA	mmu-miR-16_st
mmu-mir-22	1,3	0,0154	0,2961	miRNA	mmu-miR-22_st
mmu-mir-30c-1	1,3	0,0432	0,3631	stem-loop	hp_mmu-mir-30c-1_st
mmu-mir-452	1,3	0,0270	0,3116	miRNA	mmu-miR-452_st
mmu-miR-683	1,3	0,0155	0,2961	miRNA	mmu-miR-683_st
mmu-mir-1893	1,2	0,0216	0,3009	stem-loop	hp_mmu-mir-1893_st
mmu-mir-143	1,2	0,0189	0,2961	stem-loop	hp_mmu-mir-143_st
mmu-mir-106b	1,2	0,0369	0,3478	stem-loop	hp_mmu-mir-106b_st
mmu-mir-485	1,2	0,0421	0,3631	stem-loop	hp_mmu-mir-485_st
mmu-mir-883b	1,2	0,0457	0,3631	stem-loop	hp_mmu-mir-883b_st
mmu-mir-721	1,2	0,0469	0,3646	miRNA	mmu-miR-721_st
mmu-mir-19b-1	1,2	0,0316	0,3254	stem-loop	hp_mmu-mir-19b-1_x_st
mmu-mir-293	1,1	0,0387	0,3574	miRNA*	mmu-miR-293-star_st
mmu-mir-129-1	1,1	0,0345	0,3478	stem-loop	hp_mmu-mir-129-1_st
mmu-mir-1929	-1,1	0,0456	0,3631	miRNA	mmu-miR-1929_st
mmu-miR-465c-3p	-1,2	0,0297	0,3168	miRNA	mmu-miR-465c-3p_st
mmu-mir-293	-1,2	0,0045	0,2737	miRNA	mmu-miR-293_st
mmu-mir-16-2	-1,2	0,0461	0,3637	stem-loop	hp_mmu-mir-16-2_st
mmu-mir-146a	-1,2	0,0204	0,2961	stem-loop	hp_mmu-mir-146a_x_st
mmu-miR-125b-5p	-1,2	0,0051	0,2737	miRNA	mmu-miR-125b-5p_st
mmu-mir-667	-1,2	0,0496	0,3731	stem-loop	hp_mmu-mir-667_st
mmu-mir-574	-1,2	0,0456	0,3631	stem-loop	hp_mmu-mir-574_st
mmu-mir-292	-1,2	0,0437	0,3631	stem-loop	hp_mmu-mir-292_st
mmu-mir-710	-1,2	0,0297	0,3168	stem-loop	hp_mmu-mir-710_st
mmu-mir-33	-1,2	0,0193	0,2961	stem-loop	hp_mmu-mir-33_st
mmu-mir-125b-2	-1,2	0,0221	0,3029	stem-loop	hp_mmu-mir-125b-2_x_st
mmu-mir-3470a	-1,2	0,0009	0,1704	stem-loop	hp_mmu-mir-3470a_x_st
mmu-mir-294	-1,2	0,0269	0,3116	miRNA*	mmu-miR-294-star_st
mmu-mir-377	-1,2	0,0050	0,2737	stem-loop	hp_mmu-mir-377_st
mmu-mir-1953	-1,2	0,0103	0,2916	stem-loop	hp_mmu-mir-1953_st
mmu-miR-129-3p	-1,2	0,0418	0,3631	miRNA	mmu-miR-129-3p_st
mmu-mir-107	-1,2	0,0397	0,3631	stem-loop	hp_mmu-mir-107_x_st

mmu-mir-200a	-1,2	0,0262	0,3116	stem-loop	hp_mmu-mir-200a_st
mmu-mir-544	-1,2	0,0428	0,3631	miRNA	mmu-miR-544_st
mmu-mir-599	-1,3	0,0431	0,3631	miRNA	mmu-miR-599_st
mmu-mir-9-1	-1,3	0,0282	0,3146	stem-loop	hp_mmu-mir-9-1_x_st
mmu-mir-466e-5p	-1,3	0,0053	0,2737	miRNA	mmu-miR-466e-5p_st
mmu-mir-9-2	-1,3	0,0041	0,2737	stem-loop	hp_mmu-mir-9-2_x_st
mmu-mir-683-2	-1,3	0,0199	0,2961	stem-loop	hp_mmu-mir-683-2_s_st
mmu-mir-509	-1,3	0,0239	0,3116	miRNA	mmu-miR-509-3p_st
mmu-mir-151	-1,3	0,0469	0,3646	stem-loop	hp_mmu-mir-151_st
mmu-mir-467e	-1,3	0,0338	0,3437	miRNA*	mmu-miR-467e-star_st
mmu-mir-376b	-1,3	0,0360	0,3478	stem-loop	hp_mmu-mir-376b_st
mmu-mir-676	-1,3	0,0058	0,2737	miRNA	mmu-miR-676_st
mmu-mir-135b	-1,3	0,0022	0,2399	stem-loop	hp_mmu-mir-135b_st
mmu-mir-758	-1,3	0,0195	0,2961	miRNA	mmu-miR-758_st
mmu-mir-465a	-1,3	0,0450	0,3631	stem-loop	hp_mmu-mir-465a_x_st
mmu-mir-217	-1,3	0,0196	0,2961	miRNA	mmu-miR-217_st
mmu-mir-125a	-1,3	0,0276	0,3141	stem-loop	hp_mmu-mir-125a_st
mmu-mir-767	-1,4	0,0051	0,2737	stem-loop	hp_mmu-mir-767_st
mmu-mir-135a-1	-1,4	0,0021	0,2399	stem-loop	hp_mmu-mir-135a-1_st
mmu-mir-875	-1,4	0,0448	0,3631	miRNA	mmu-miR-875-3p_st
mmu-mir-376b	-1,4	0,0446	0,3631	miRNA*	mmu-miR-376b-star_st
mmu-mir-30d	-1,4	0,0111	0,2961	miRNA	mmu-miR-30d_st
mmu-mir-101b	-1,4	0,0135	0,2961	stem-loop	hp_mmu-mir-101b_st
mmu-mir-138-2	-1,4	0,0148	0,2961	stem-loop	hp_mmu-mir-2136_st
mmu-let-7f-2	-1,4	0,0444	0,3631	stem-loop	hp_mmu-let-7f-2_x_st
mmu-mir-18b	-1,5	0,0413	0,3631	miRNA	mmu-miR-18b_st
mmu-mir-466g	-1,5	0,0035	0,2737	stem-loop	hp_mmu-mir-466g_st
mmu-mir-1937b-5 (#)	-1,5	0,0040	0,2737	stem-loop/tRNA	hp_mmu-mir-1937b-5_s_st
mmu-mir-340	-1,5	0,0253	0,3116	stem-loop	hp_mmu-mir-340_st
mmu-mir-2136	-1,5	0,0005	0,1627	stem-loop	hp_mmu-mir-2136_st
mmu-mir-208a	-1,5	0,0029	0,2737	stem-loop	hp_mmu-mir-208a_st
mmu-miR-30c	-1,5	0,0436	0,3631	miRNA	mmu-miR-30c_st
mmu-miR-133a	-1,6	0,0260	0,3116	miRNA	mmu-miR-133a_st
mmu-mir-677	-1,7	0,0297	0,3168	stem-loop	hp_mmu-mir-677_st
mmu-mir-743b	-1,7	0,0065	0,2748	miRNA	mmu-miR-743b-3p_st
mmu-mir-149	-1,7	0,0496	0,3731	miRNA	mmu-miR-149_st
mmu-mir-410	-1,7	0,0249	0,3116	miRNA	mmu-miR-410_st
mmu-mir-1983	-1,7	0,0126	0,2961	stem-loop	hp_mmu-mir-1983_st
mmu-mir-875	-1,7	0,0267	0,3116	stem-loop	hp_mmu-mir-875_st
mmu-mir-339	-1,8	0,0470	0,3646	miRNA	mmu-miR-339-5p_st
mmu-mir-99b	-1,8	0,0064	0,2748	miRNA	mmu-miR-99b_st
mmu-mir-181a-2	-1,9	0,0458	0,3631	stem-loop	hp_mmu-mir-181a-2_st
mmu-mir-1274a	-1,9	0,0020	0,2399	miRNA/tRNA1	mmu-miR-1274a_st
mmu-miR-3473	-1,9	0,0195	0,2961	miRNA	mmu-miR-3473_st
mmu-mir-351	-1,9	0,0170	0,2961	miRNA	mmu-miR-351_st
mmu-mir-125a	-2,1	0,0121	0,2961	miRNA	mmu-miR-125a-5p_st
mmu-mir-669l	-2,1	0,0148	0,2961	stem-loop	hp_mmu-mir-669l_st
v11_mmu-miR-805 (%)	-2,2	0,0206	0,2961	miRNA/mtDNA	v11_mmu-miR-805_st
mmu-mir-877	-2,4	0,0189	0,2961	miRNA	mmu-miR-877_st
mmu-mir-145	-2,4	0,0230	0,3088	miRNA	mmu-miR-145_st
mmu-mir-465c-2	-2,8	0,0361	0,3478	stem-loop	hp_mmu-mir-465c-2_s_st
mmu-mir-465c-1	-2,8	0,0271	0,3116	stem-loop	hp_mmu-mir-465c-1_s_st
mmu-mir-2146 ($)	-2,9	0,0114	0,2961	miRNA/rRNA	mmu-miR-2146_st
mmu-miR-466f-3p	-2,9	0,0406	0,3631	miRNA	mmu-miR-466f-3p_st
mmu-mir-3072	-3,1	0,0314	0,3254	miRNA*	mmu-miR-3072-star_st
mmu-mir-1892	-3,3	0,0070	0,2748	miRNA	mmu-miR-1892_st
mmu-mir-1895	-4,2	0,0184	0,2961	miRNA	mmu-miR-1895_st
mmu-miR-2145 ($)	-4,4	0,0106	0,2941	miRNA/rRNA	mmu-miR-2145_st
mmu-mir-1937a (#)	-5,1	0,0173	0,2961	miRNA/tRNA	mmu-miR-1937a_st
mmu-mir-1959 (#)	-6,9	0,0009	0,1704	miRNA/tRNA	mmu-miR-1959_st

(&) Originally named miR-1980, this sequence maps to both miR-1839 and to the snoRNA SCARNA15 (ACA45, Ender et al., 2008, PMID:190267820)

(#) The sequence is a fragment of tRNA, and is therefore not a likely to be a bona fide miRNA (Schopman et al., 2010, PMID:20818168).

(%) The sequence maps to the mouse mitochondrial (mt) genome

($) The sequence is a fragment of rRNA (Chiang et al, 2010, PMID:20413612)

Note: The q-value of an individual hypothesis test is the maximum FDR at which the test may be called significant.
