# Supplementary material for: MicroRNA-206: A Potential Circulating Biomarker Candidate for Amyotrophic Lateral Sclerosis
Source: PLoS One. 2014 Feb 20;9(2):e89065. doi: 10.1371/journal.pone.0089065 (PMC3930686; doi:10.1371/journal.pone.0089065)
Supplement: Table S2 — Microarray changes in SOD1-G93A SOL muscle compared with wild type SOL. All significant changes without multiple corrections are listed. Positive fold change (FC) indicates higher expression in the SOD1-G93A mutants, and negative FC lower higher expression in the wild type animals. Probeset ID refers to the Affymetrix probeset identifier. (PDF) [file pone.0089065.s007.pdf]

Transcript ID	FC (SOL+ vs. SOL-)	p-value	FDR(q-value)	Sequence Type	Probeset ID
mmu-mir-675	2,7	0,0441	0,9906	miRNA	mmu-miR-675-3p_st
mmu-mir-215	1,4	0,0175	0,9906	miRNA	mmu-miR-215_st
mmu-mir-496	1,4	0,0230	0,9906	stem-loop	hp_mmu-mir-496_st
mmu-mir-574	1,4	0,0034	0,9906	stem-loop	hp_mmu-mir-574_st
mmu-mir-125b-1	1,3	0,0212	0,9906	stem-loop	hp_mmu-mir-125b-1_x_st
mmu-mir-495	1,3	0,0233	0,9906	stem-loop	hp_mmu-mir-495_st
mmu-mir-412	1,3	0,0376	0,9906	stem-loop	hp_mmu-mir-412_st
mmu-mir-744	1,3	0,0080	0,9906	miRNA*	mmu-miR-744-star_st
mmu-mir-451	1,3	0,0159	0,9906	stem-loop	hp_mmu-mir-451_st
mmu-mir-128-2	1,3	0,0500	0,9906	stem-loop	hp_mmu-mir-128-2_x_st
mmu-mir-509	1,3	0,0375	0,9906	miRNA	mmu-miR-509-3p_st
mmu-mir-302b	1,3	0,0015	0,9906	stem-loop	hp_mmu-mir-302b_st
mmu-mir-883b	1,3	0,0163	0,9906	stem-loop	hp_mmu-mir-883b_st
mmu-mir-1896	1,2	0,0348	0,9906	stem-loop	hp_mmu-mir-1896_st
mmu-mir-2136	1,2	0,0184	0,9906	stem-loop	hp_mmu-mir-2136_st
mmu-let-7a-star	1,2	0,0287	0,9906	miRNA*	mmu-let-7a-star_st
mmu-mir-377	1,1	0,0384	0,9906	stem-loop	hp_mmu-mir-377_st
mmu-miR-125b-5p	1,1	0,0237	0,9906	miRNA	mmu-miR-125b-5p_st
mmu-mir-687	1,1	0,0376	0,9906	miRNA	mmu-miR-687_st
mmu-mir-687	-1,2	0,0431	0,9906	stem-loop	hp_mmu-mir-687_st
mmu-mir-138-1	-1,2	0,0293	0,9906	stem-loop	hp_mmu-mir-138-1_st
mmu-mir-721	-1,2	0,0377	0,9906	miRNA	mmu-miR-721_st
mmu-mir-2139	-1,2	0,0257	0,9906	stem-loop	hp_mmu-mir-2139_st
mmu-mir-30c-1	-1,2	0,0194	0,9906	stem-loop	hp_mmu-mir-30c-1_x_st
mmu-mir-378	-1,2	0,0212	0,9906	stem-loop	hp_mmu-mir-378_st
mmu-mir-467e	-1,2	0,0364	0,9906	miRNA	mmu-miR-467e_st
mmu-mir-1938	-1,3	0,0265	0,9906	stem-loop	hp_mmu-mir-1938_st
mmu-let-7g	-1,3	0,0306	0,9906	stem-loop	hp_mmu-let-7g_st
mmu-mir-143	-1,3	0,0095	0,9906	stem-loop	hp_mmu-mir-143_st
mmu-mir-871	-1,3	0,0480	0,9906	stem-loop	hp_mmu-mir-871_st
mmu-mir-1970	-1,3	0,0088	0,9906	stem-loop	hp_mmu-mir-1970_st
mmu-mir-376b	-1,3	0,0314	0,9906	stem-loop	hp_mmu-mir-376b_st
mmu-mir-181a-1	-1,4	0,0138	0,9906	stem-loop	hp_mmu-mir-181a-1_st
mmu-mir-3099	-1,4	0,0370	0,9906	stem-loop	hp_mmu-mir-3099_st
mmu-mir-15b	-1,4	0,0011	0,9906	miRNA*	mmu-miR-15b-star_st
mmu-mir-466k	-1,4	0,0187	0,9906	stem-loop	hp_mmu-mir-466k_st
mmu-mir-195	-1,4	0,0099	0,9906	stem-loop	hp_mmu-mir-195_st
mmu-let-7f-1	-1,5	0,0200	0,9906	stem-loop	hp_mmu-let-7f-1_x_st
mmu-mir-204	-1,5	0,0101	0,9906	miRNA	mmu-miR-204_st
mmu-mir-188	-1,6	0,0366	0,9906	stem-loop	hp_mmu-mir-188_st
mmu-mir-324	-1,8	0,0295	0,9906	miRNA	mmu-miR-324-3p_st
mmu-mir-207	-2,6	0,0090	0,9906	miRNA	mmu-miR-207_st

Note: The q-value of an individual hypothesis test is the maximum FDR at which the test may be called significant.
