# Supplementary material for: MicroRNA-206: A Potential Circulating Biomarker Candidate for Amyotrophic Lateral Sclerosis
Source: PLoS One. 2014 Feb 20;9(2):e89065. doi: 10.1371/journal.pone.0089065 (PMC3930686; doi:10.1371/journal.pone.0089065)
Supplement: Table S3 — Preferential expression of microarray probesets in wild type EDL vs wild type SOL. All significant changes without multiple corrections are listed. Positive fold change (FC) indicates preferential (higher) expression in the wild type EDL, and negative FC lower preferential expression in the wild type SOL. Probeset ID refers to the Affymetrix probeset identifier. (PDF) [file pone.0089065.s008.pdf]

Transcript ID	FC (EDL- vs. S	p-value	FDR(q-value)	Sequence Type	Probeset ID	mSOD1 EDL
mmu-miR-1937b (#)	8,1	0,0117	0,4599	miRNA/tRNA	mmu-miR-1937b_st	↓
mmu-mir-1959 (#)	7,6	0,0160	0,4699	miRNA/tRNA	mmu-miR-1959_st	↓
mmu-mir-1937a (#)	5,3	0,0441	0,5262	miRNA/tRNA	mmu-miR-1937a_st	↑
mmu-mir-193	4,5	0,0215	0,4728	miRNA*	mmu-miR-193-star_st	
mmu-miR-714	4,2	0,0352	0,5012	miRNA	mmu-miR-714_st	
mmu-miR-3473	3,8	0,0010	0,2730	miRNA	mmu-miR-3473_st	↓
mmu-mir-181a-2	3,7	0,0031	0,3590	stem-loop	hp_mmu-mir-181a-2_st	↓
mmu-mir-149	3,3	0,0087	0,4495	miRNA	mmu-miR-149_st	↓
mmu-mir-486	3,0	0,0322	0,5012	miRNA	mmu-miR-486_st	
mmu-mir-181a-2	2,9	0,0194	0,4728	stem-loop	hp_mmu-mir-181a-2_x_st	
v11_mmu-miR-805 (%)	2,6	0,0194	0,4728	miRNA/mtDNA	v11_mmu-miR-805_st	↓
mmu-miR-133a	2,3	0,0245	0,4809	miRNA	mmu-miR-133a_st	↓
mmu-miR-92a	2,3	0,0425	0,5222	miRNA	mmu-miR-92a_st	
mmu-mir-877	2,2	0,0008	0,2730	miRNA	mmu-miR-877_st	↓
mmu-mir-705	2,2	0,0370	0,5012	miRNA	mmu-miR-705_st	
mmu-mir-149	2,2	0,0002	0,2730	stem-loop	hp_mmu-mir-149_st	
mmu-miR-181a	1,9	0,0194	0,4728	miRNA	mmu-miR-181a_st	
mmu-mir-133b	1,9	0,0060	0,4495	miRNA	mmu-miR-133b_st	
mmu-mir-125a	1,8	0,0350	0,5012	miRNA	mmu-miR-125a-5p_st	↓
mmu-mir-331	1,7	0,0325	0,5012	stem-loop	hp_mmu-mir-331_st	
mmu-mir-695	1,7	0,0390	0,5012	miRNA	mmu-miR-695_st	
mmu-mir-677	1,7	0,0324	0,5012	stem-loop	hp_mmu-mir-677_st	↓
mmu-mir-138-2	1,7	0,0301	0,5012	stem-loop	hp_mmu-mir-138-2_x_st	↓
mmu-mir-2136	1,6	0,0061	0,4495	stem-loop	hp_mmu-mir-2136_st	↓
mmu-mir-483	1,6	0,0378	0,5012	miRNA*	mmu-miR-483-star_st	
mmu-mir-100	1,5	0,0348	0,5012	miRNA	mmu-miR-100_st	
mmu-mir-676	1,5	0,0016	0,3258	miRNA	mmu-miR-676_st	↓
mmu-mir-208a	1,5	0,0121	0,4599	stem-loop	hp_mmu-mir-208a_st	↓
mmu-mir-200a	1,5	0,0186	0,4728	stem-loop	hp_mmu-mir-200a_st	↓
mmu-mir-744	1,4	0,0062	0,4495	miRNA*	mmu-miR-744-star_st	
mmu-mir-377	1,4	0,0069	0,4495	stem-loop	hp_mmu-mir-377_st	↓
mmu-mir-466d	1,4	0,0109	0,4599	miRNA	mmu-miR-466d-3p_st	
mmu-mir-1965	1,4	0,0204	0,4728	stem-loop	hp_mmu-mir-1965_st	
mmu-mir-378	1,4	0,0286	0,5012	miRNA*	mmu-miR-378-star_st	
mmu-mir-201	1,4	0,0232	0,4746	stem-loop	hp_mmu-mir-201_st	
mmu-mir-1957	1,3	0,0125	0,4599	stem-loop	hp_mmu-mir-1957_st	
mmu-mir-135b	1,3	0,0289	0,5012	stem-loop	hp_mmu-mir-135b_st	↓
mmu-mir-509	1,3	0,0302	0,5012	miRNA	mmu-miR-509-3p_st	↓
mmu-mir-346	1,3	0,0086	0,4495	stem-loop	hp_mmu-mir-346_st	
mmu-mir-767	1,3	0,0089	0,4495	stem-loop	hp_mmu-mir-767_st	↓
mmu-mir-449c	1,3	0,0124	0,4599	miRNA	mmu-miR-449c_st	
mmu-miR-465c-3p	1,3	0,0325	0,5012	miRNA	mmu-miR-465c-3p_st	↓
mmu-mir-710	1,3	0,0170	0,4728	stem-loop	hp_mmu-mir-710_st	↓
mmu-mir-124-1	1,3	0,0348	0,5012	stem-loop	hp_mmu-mir-124-1_s_st	
mmu-mir-466e	1,2	0,0228	0,4728	miRNA	mmu-miR-466e-5p_st	↓
mmu-miR-129-3p	1,2	0,0036	0,3925	miRNA	mmu-miR-129-3p_st	↓
mmu-mir-146b	1,2	0,0089	0,4495	miRNA*	mmu-miR-146b-star_st	
mmu-mir-217	1,2	0,0436	0,5260	miRNA	mmu-miR-217_st	↓
mmu-mir-465b-1	1,2	0,0227	0,4728	stem-loop	hp_mmu-mir-465b-1_x_st	
mmu-mir-194-2	1,2	0,0405	0,5089	stem-loop	hp_mmu-mir-194-2_x_st	
mmu-mir-93	1,2	0,0422	0,5222	miRNA*	mmu-miR-93-star_st	
mmu-mir-2136	1,2	0,0358	0,5012	miRNA	mmu-miR-2136_st	↓
mmu-mir-341	1,2	0,0350	0,5012	stem-loop	hp_mmu-mir-341_st	
mmu-mir-455	1,2	0,0128	0,4599	stem-loop	hp_mmu-mir-455_st	
mmu-mir-203	1,2	0,0140	0,4599	miRNA*	mmu-miR-203-star_st	
mmu-mir-693	1,2	0,0039	0,3925	stem-loop	hp_mmu-mir-693_st	
mmu-miR-125b-5p	1,2	0,0010	0,2730	miRNA	mmu-miR-125b-5p_st	↓
mmu-mir-465b-1	1,2	0,0140	0,4599	stem-loop	hp_mmu-mir-465b-1_s_st	
mmu-mir-883b	1,2	0,0353	0,5012	miRNA	mmu-miR-883b-3p_st	
mmu-mir-291a	1,1	0,0443	0,5262	miRNA	mmu-miR-291a-5p_st	
mmu-miR-181a-1-star	1,1	0,0267	0,4957	miRNA*	mmu-miR-181a-1-star_st	
mmu-mir-3099	1,1	0,0149	0,4599	miRNA	mmu-miR-3099_st	
mmu-mir-1970	-1,0	0,0026	0,3590	stem-loop	hp_mmu-mir-1970_st	
mmu-mir-299	-1,1	0,0019	0,3377	stem-loop	hp_mmu-mir-299_x_st	

mmu-mir-704	-1,1	0,0399	0,5080	stem-loop	hp_mmu-mir-704_st	
mmu-mir-694	-1,1	0,0116	0,4599	stem-loop	hp_mmu-mir-694_st	
mmu-mir-30b	-1,1	0,0144	0,4599	stem-loop	hp_mmu-mir-30b_st	
mmu-mir-24-2	-1,1	0,0149	0,4599	stem-loop	hp_mmu-mir-24-2_x_st	
mmu-mir-669h	-1,1	0,0028	0,3590	stem-loop	hp_mmu-mir-669h_x_st	
mmu-mir-505	-1,1	0,0082	0,4495	stem-loop	hp_mmu-mir-505_st	
mmu-mir-30a	-1,2	0,0380	0,5012	stem-loop	hp_mmu-mir-30a_st	
mmu-miR-124	-1,2	0,0389	0,5012	miRNA	mmu-miR-124_st	
mmu-mir-669d	-1,2	0,0093	0,4512	stem-loop	hp_mmu-mir-669d_st	
mmu-mir-133a-2	-1,2	0,0274	0,4957	stem-loop	hp_mmu-mir-133a-2_st	
mmu-mir-338	-1,2	0,0173	0,4728	stem-loop	hp_mmu-mir-338_st	
mmu-miR-712	-1,2	0,0271	0,4957	miRNA	mmu-miR-712_st	
mmu-mir-676	-1,2	0,0384	0,5012	miRNA*	mmu-miR-676-star_st	
mmu-mir-455	-1,2	0,0321	0,5012	miRNA*	mmu-miR-455-star_st	
mmu-mir-22	-1,3	0,0155	0,4658	miRNA	mmu-miR-22_st	↑
mmu-mir-466h	-1,3	0,0382	0,5012	stem-loop	hp_mmu-mir-466h_x_st	↑
mmu-mir-764	-1,3	0,0084	0,4495	miRNA	mmu-miR-764-5p_st	
mmu-mir-330	-1,3	0,0472	0,5458	stem-loop	hp_mmu-mir-330_st	
mmu-mir-143	-1,3	0,0052	0,4495	stem-loop	hp_mmu-mir-143_st	↑
mmu-mir-323	-1,3	0,0206	0,4728	stem-loop	hp_mmu-mir-323_st	↑
mmu-mir-21	-1,3	0,0255	0,4870	miRNA*	mmu-miR-21-star_st	↑
mmu-mir-203	-1,3	0,0012	0,2730	stem-loop	hp_mmu-mir-203_st	
mmu-mir-451	-1,3	0,0079	0,4495	stem-loop	hp_mmu-mir-451_x_st	
mmu-mir-1937a (#)	-1,3	0,0149	0,4599	stem-loop/tRNA	hp_mmu-mir-1937a_st	↑
mmu-mir-466f-4	-1,4	0,0111	0,4599	stem-loop	hp_mmu-mir-466f-4_x_st	
mmu-mir-764	-1,4	0,0121	0,4599	stem-loop	hp_mmu-mir-764_st	
mmu-mir-721	-1,4	0,0215	0,4728	miRNA	mmu-miR-721_st	↑
mmu-mir-467c	-1,4	0,0150	0,4599	miRNA	mmu-miR-467c_st	
mmu-mir-467e	-1,4	0,0291	0,5012	miRNA	mmu-miR-467e_st	↑
mmu-mir-206	-1,4	0,0186	0,4728	stem-loop	hp_mmu-mir-206_st	
mmu-mir-93	-1,4	0,0271	0,4957	miRNA	mmu-miR-93_st	↑
mmu-mir-1954	-1,5	0,0430	0,5236	miRNA	mmu-miR-1954_st	↑
mmu-mir-23a	-1,5	0,0249	0,4809	stem-loop	hp_mmu-mir-23a_x_st	↑
mmu-mir-148a	-1,6	0,0315	0,5012	miRNA	mmu-miR-148a_st	↑
mmu-mir-204	-1,8	0,0378	0,5012	miRNA	mmu-miR-204_st	↑
mmu-mir-467d	-1,8	0,0050	0,4495	miRNA*	mmu-miR-467d-star_st	
mmu-mir-34b	-1,8	0,0210	0,4728	miRNA	mmu-miR-34b-3p_st	
mmu-let-7e	-1,9	0,0086	0,4495	miRNA	mmu-let-7e_st	
mmu-mir-411	-1,9	0,0223	0,4728	miRNA	mmu-miR-411_st	
mmu-mir-127	-2,0	0,0407	0,5089	miRNA*	mmu-miR-127-star_st	
mmu-mir-199b	-2,0	0,0238	0,4792	miRNA	mmu-miR-199b_st	
mmu-mir-1941	-2,1	0,0471	0,5458	miRNA	mmu-miR-1941-5p_st	↑
mmu-mir-17	-2,1	0,0369	0,5012	miRNA	mmu-miR-17_st	↑
mmu-mir-23a	-2,2	0,0247	0,4809	stem-loop	hp_mmu-mir-23a_st	↑
mmu-mir-27b	-2,2	0,0227	0,4728	miRNA	mmu-miR-27b_st	↑
mmu-miR-24	-2,3	0,0187	0,4728	miRNA	mmu-miR-24_st	↑
mmu-mir-208b	-2,6	0,0367	0,5012	miRNA	mmu-miR-208b_st	
mmu-mir-421	-2,7	0,0376	0,5012	miRNA	mmu-miR-421_st	↑
mmu-mir-425	-2,9	0,0129	0,4599	miRNA*	mmu-miR-425-star_st	↑
mmu-mir-500	-3,7	0,0004	0,2730	miRNA	mmu-miR-500_st	↑
mmu-mir-675	-4,1	0,0493	0,5663	miRNA	mmu-miR-675-3p_st	
mmu-mir-20b	-5,5	0,0025	0,3590	miRNA	mmu-miR-20b_st	↑
mmu-miR-24-2-star	-5,9	0,0371	0,5012	miRNA*	mmu-miR-24-2-star_st	↑
mmu-mir-27a	-6,8	0,0462	0,5439	miRNA*	mmu-miR-27a-star_st	
mmu-mir-322/424	-7,6	0,0390	0,5012	miRNA	mmu-miR-322_st	↑
mmu-mir-708	-8,5	0,0200	0,4728	miRNA	mmu-miR-708_st	↑
mmu-mir-671	-9,1	0,0180	0,4728	miRNA	mmu-miR-671-5p_st	↑
mmu-mir-451	-9,1	0,0329	0,5012	miRNA	mmu-miR-451_st	↑
mmu-mir-206	-10,9	0,0209	0,4728	miRNA	mmu-miR-206_st	↑

(#) The sequence is a fragment of tRNA, and is therefore not a likely to be a bona fide miRNA (Schopman et al., 2010, PMID:20818168).

(%) The sequence maps to the mouse mitochondrial (mt) genome

Note: The q-value of an individual hypothesis test is the maximum FDR at which the test may be called significant.
