# Supplementary material for: MicroRNA-206: A Potential Circulating Biomarker Candidate for Amyotrophic Lateral Sclerosis
Source: PLoS One. 2014 Feb 20;9(2):e89065. doi: 10.1371/journal.pone.0089065 (PMC3930686; doi:10.1371/journal.pone.0089065)
Supplement: Table S4 — Preferential expression of microarray probesets in SOD1-G93A EDL vs SOD1-G93A SOL. All significant changes without multiple corrections are listed. Positive fold change (FC) indicates preferential (higher) expression in the SOD1-G93A EDL, and negative FC lower preferential expression in the SOD1-G93A SOL. Probeset ID refers to the Affymetrix probeset identifier. (PDF) [file pone.0089065.s009.pdf]

Transcript ID	FC (EDL+ vs.SOL+)	p-value	FDR(q-value)	Sequence Type	Probeset ID
mmu-mir-193	5,4	0,0332	0,7843	miRNA*	mmu-miR-193-star_st
mmu-miR-138	5,1	0,0092	0,7166	miRNA	mmu-miR-138_st
mmu-mir-433	3,3	0,0066	0,7166	miRNA	mmu-miR-433_st
mmu-mir-486	2,4	0,0433	0,7843	miRNA	mmu-miR-486_st
mmu-mir-29c	2,2	0,0001	0,0898	miRNA	mmu-miR-29c_st
mmu-mir-181a-2	2,0	0,0414	0,7843	stem-loop	hp_mmu-mir-181a-2_st
mmu-mir-598	1,8	0,0492	0,7843	miRNA	mmu-miR-598_st
mmu-mir-28	1,7	0,0411	0,7843	miRNA*	mmu-miR-28-star_st
mmu-mir-700	1,7	0,0228	0,7843	miRNA	mmu-miR-700_st
mmu-mir-1198	1,5	0,0457	0,7843	miRNA	mmu-miR-1198_st
mmu-mir-598	1,4	0,0496	0,7843	stem-loop	hp_mmu-mir-598_st
mmu-miR-7a-star	1,4	0,0436	0,7843	miRNA*	mmu-miR-7a-star_st
mmu-mir-183	1,4	0,0360	0,7843	miRNA	mmu-miR-183_st
mmu-mir-361	1,4	0,0404	0,7843	miRNA	mmu-miR-361_st
mmu-mir-433	1,3	0,0290	0,7843	stem-loop	hp_mmu-mir-433_st
mmu-mir-222	1,3	0,0393	0,7843	miRNA	mmu-miR-222_st
mmu-mir-106b	1,3	0,0368	0,7843	stem-loop	hp_mmu-mir-106b_st
mmu-mir-467g	1,3	0,0340	0,7843	miRNA	mmu-miR-467g_st
mmu-mir-210	1,3	0,0487	0,7843	miRNA	mmu-miR-210_st
mmu-mir-466k	1,3	0,0216	0,7843	stem-loop	hp_mmu-mir-466k_st
mmu-mir-15b	1,3	0,0033	0,7166	miRNA*	mmu-miR-15b-star_st
mmu-mir-709	1,3	0,0397	0,7843	miRNA	mmu-miR-709_st
mmu-mir-376b	1,3	0,0023	0,7166	miRNA*	mmu-miR-376b-star_st
mmu-mir-291a	1,3	0,0078	0,7166	stem-loop	hp_mmu-mir-291a_x_st
mmu-let-7b	1,2	0,0355	0,7843	miRNA	mmu-let-7b_st
mmu-mir-666	1,2	0,0089	0,7166	miRNA	mmu-miR-666-3p_st
mmu-mir-467h	1,2	0,0066	0,7166	stem-loop	hp_mmu-mir-467h_x_st
mmu-mir-1901	1,2	0,0177	0,7843	stem-loop	hp_mmu-mir-1901_st
mmu-mir-880	1,2	0,0250	0,7843	miRNA	mmu-miR-880_st
mmu-mir-187	1,2	0,0049	0,7166	stem-loop	hp_mmu-mir-187_st
mmu-mir-200b	1,2	0,0159	0,7843	miRNA	mmu-miR-200b_st
mmu-mir-700	1,2	0,0253	0,7843	stem-loop	hp_mmu-mir-700_st
mmu-mir-1937b-3 (#)	1,1	0,0128	0,7467	stem-loop	hp_mmu-mir-1937b-3_st
mmu-mir-1904	1,1	0,0086	0,7166	stem-loop	hp_mmu-mir-1904_st
mmu-mir-92a-1	1,1	0,0195	0,7843	stem-loop	hp_mmu-mir-92a-1_x_st
mmu-mir-154	1,1	0,0364	0,7843	stem-loop	hp_mmu-mir-154_st
mmu-mir-2145-1	1,1	0,0288	0,7843	stem-loop	hp_mmu-mir-2145-1_st
mmu-mir-1190	1,1	0,0117	0,7166	stem-loop	hp_mmu-mir-1190_st
mmu-mir-30a	1,1	0,0109	0,7166	stem-loop	hp_mmu-mir-30a_st
mmu-mir-1933	1,1	0,0196	0,7843	miRNA	mmu-miR-1933-5p_st
mmu-mir-146b	1,1	0,0429	0,7843	stem-loop	hp_mmu-mir-146b_st
mmu-mir-465b-2	1,1	0,0498	0,7843	stem-loop	hp_mmu-mir-465b-2_x_st
mmu-mir-1963	1,0	0,0112	0,7166	miRNA	mmu-miR-1963_st
mmu-mir-3474	-1,1	0,0243	0,7843	stem-loop	hp_mmu-mir-3474_st
mmu-mir-142	-1,1	0,0425	0,7843	miRNA	mmu-miR-142-5p_st
mmu-mir-19a	-1,1	0,0245	0,7843	stem-loop	hp_mmu-mir-19a_st
mmu-mir-453	-1,1	0,0399	0,7843	stem-loop	hp_mmu-mir-453_st
mmu-miR-125b-5p	-1,1	0,0133	0,7467	miRNA	mmu-miR-125b-5p_st
mmu-mir-432	-1,2	0,0336	0,7843	stem-loop	hp_mmu-mir-432_st
mmu-mir-466c	-1,2	0,0408	0,7843	stem-loop	hp_mmu-mir-466c_x_st
mmu-mir-141	-1,2	0,0108	0,7166	stem-loop	hp_mmu-mir-141_x_st
mmu-mir-302b	-1,2	0,0308	0,7843	stem-loop	hp_mmu-mir-302b_st
mmu-mir-882	-1,2	0,0239	0,7843	stem-loop	hp_mmu-mir-882_st
mmu-mir-23a	-1,2	0,0231	0,7843	stem-loop	hp_mmu-mir-23a_x_st
mmu-mir-23b	-1,2	0,0115	0,7166	stem-loop	hp_mmu-mir-23b_x_st
mmu-mir-1953	-1,2	0,0044	0,7166	stem-loop	hp_mmu-mir-1953_st
mmu-mir-1958	-1,2	0,0069	0,7166	stem-loop	hp_mmu-mir-1958_st
mmu-mir-509	-1,2	0,0298	0,7843	miRNA	mmu-miR-509-3p_st
mmu-mir-468	-1,3	0,0073	0,7166	miRNA	mmu-miR-468_st
mmu-miR-712-star	-1,3	0,0234	0,7843	miRNA*	mmu-miR-712-star_st
mmu-mir-212	-1,3	0,0191	0,7843	stem-loop	hp_mmu-mir-212_st
mmu-mir-467e	-1,3	0,0296	0,7843	miRNA*	mmu-miR-467e-star_st
mmu-mir-132	-1,3	0,0257	0,7843	miRNA	mmu-miR-132_st
mmu-mir-574	-1,3	0,0224	0,7843	stem-loop	hp_mmu-mir-574_st

mmu-let-7a-star	-1,3	0,0096	0,7166	miRNA*	mmu-let-7a-star_st
mmu-mir-140	-1,3	0,0107	0,7166	miRNA*	mmu-miR-140-star_st
mmu-miR-16	-1,3	0,0137	0,7467	miRNA	mmu-miR-16_st
mmu-mir-30b	-1,4	0,0288	0,7843	miRNA	mmu-miR-30b_st
mmu-mir-130a	-1,4	0,0309	0,7843	miRNA	mmu-miR-130a_st
mmu-mir-15b	-1,4	0,0100	0,7166	miRNA	mmu-miR-15b_st
mmu-mir-207	-1,4	0,0272	0,7843	stem-loop	hp_mmu-mir-207_st
mmu-mir-30d	-1,5	0,0250	0,7843	miRNA	mmu-miR-30d_st
mmu-mir-466i	-1,7	0,0270	0,7843	miRNA	mmu-miR-466i_st
mmu-mir-10a	-1,8	0,0420	0,7843	miRNA	mmu-miR-10a_st
mmu-mir-223	-1,9	0,0415	0,7843	miRNA	mmu-miR-223_st
mmu-mir-208b	-4,0	0,0080	0,7166	miRNA	mmu-miR-208b_st
mmu-mir-675	-4,9	0,0093	0,7166	miRNA	mmu-miR-675-3p_st

(#) The sequence is a fragment of tRNA, and is therefore not a likely to be a bona fide miRNA (Schopman et al., 2010, PMID:20818168).

Note: The q-value of an individual hypothesis test is the maximum FDR at which the test may be called significant.
